# Survival prediction landscape: an in-depth systematic literature review on activities, methods, tools, diseases, and databases

**DOI:** 10.3389/frai.2024.1428501

**Published:** 2024-07-03

**Authors:** Ahtisham Fazeel Abbasi, Muhammad Nabeel Asim, Sheraz Ahmed, Sebastian Vollmer, Andreas Dengel

**Affiliations:** ^1^Department of Computer Science, Rhineland-Palatinate Technical University of Kaiserslautern-Landau, Kaiserslautern, Germany; ^2^Smart Data & Knowledge Services, Deutsches Forschungszentrum für Künstliche Intelligenz (DFKI), Kaiserslautern, Germany

**Keywords:** survival prediction, artificial intelligence, machine learning, multiomics, cancer

## Abstract

Survival prediction integrates patient-specific molecular information and clinical signatures to forecast the anticipated time of an event, such as recurrence, death, or disease progression. Survival prediction proves valuable in guiding treatment decisions, optimizing resource allocation, and interventions of precision medicine. The wide range of diseases, the existence of various variants within the same disease, and the reliance on available data necessitate disease-specific computational survival predictors. The widespread adoption of artificial intelligence (AI) methods in crafting survival predictors has undoubtedly revolutionized this field. However, the ever-increasing demand for more sophisticated and effective prediction models necessitates the continued creation of innovative advancements. To catalyze these advancements, it is crucial to bring existing survival predictors knowledge and insights into a centralized platform. The paper in hand thoroughly examines 23 existing review studies and provides a concise overview of their scope and limitations. Focusing on a comprehensive set of 90 most recent survival predictors across 44 diverse diseases, it delves into insights of diverse types of methods that are used in the development of disease-specific predictors. This exhaustive analysis encompasses the utilized data modalities along with a detailed analysis of subsets of clinical features, feature engineering methods, and the specific statistical, machine or deep learning approaches that have been employed. It also provides insights about survival prediction data sources, open-source predictors, and survival prediction frameworks.

## 1 Introduction

According to World Health Organization (WHO), around ten thousand diseases have been discovered and each disease has unique characteristics, symptoms, and implications on human health (Haendel et al., 2020). Millions of people died from such diseases in the span of years 2000 to 2019, while cancers, cardiovascular, and infectious diseases persisted as the leading causes of mortality (Jamison, 2018; World Health Organization, 2020). Extensive research on the intersection of life and technology has yielded a wide range of therapies and medications for various well-known diseases [National Research Council (US), 2010]. However, the core idea behind traditional therapies and medications is based on the “one-size-fits-all” (Sellin, 2015). In this paradigm, a single drug is supposed to effectively treat a medical condition across a variety of patient cohorts i.e., children, old and young populations (Al-Lazikani et al., 2012; Sellin, 2015). In-depth exploration and understanding of living organisms' inherent biological processes reveal that high variability in genetics and drug responses make one-size-fits-all medication ineffective (Al-Lazikani et al., 2012; Sellin, 2015).

The groundbreaking discoveries of the factors contributing to the limited effectiveness of generalized medications marked the inception of the era of precision medicine (Ashley, 2016; Kosorok and Laber, 2019). Precision medicine offers customization in tailored medical treatments based on an individual's unique genetic makeup, and optimization in drug selection and dosage based on the individual's lifestyle, and environmental factors (Farrokhi et al., 2023). Precision medicine's adoption and effectiveness have been significantly enhanced by the accurate, cost-effective, and large-scale analysis of molecular information obtained through next-generation sequencing (Kamps et al., 2017).

In the realm of precision medicine, survival prediction plays a pivotal role in tailoring medical treatments to individual needs (Billheimer et al., 2014; Tsimberidou et al., 2019). Survival prediction categorizes patients into distinct risk groups that enhance the efficiency of resource allocation for the patients who are likely to gain the most benefit from specific treatments (Billheimer et al., 2014; Tsimberidou et al., 2019). It also enables counseling of patients and their families by predicting the expected course of the disease and potential challenges (Billheimer et al., 2014). In addition to medical treatments, survival prediction offers multiple advantages in research, particularly in the area of biomarker discovery and disease understanding (Chen et al., 2018; Sarma et al., 2020). Survival prediction models provide useful information about the correlation between different features and clinical outcomes. This correlation information enables the identification of novel biomarkers associated with disease prognosis (Sarma et al., 2020). Moreover, researchers leverage survival prediction to unravel disease heterogeneity which helps to identify distinct subtypes with different survival profiles (Hao et al., 2023). This knowledge not only aids in the stratification of homogeneous patients in clinical trials but also validates therapeutic targets by assessing their relevance in predicting patient outcomes (Glare et al., 2003). Furthermore, it enables the longitudinal monitoring of disease progression that helps to explore critical time points and progression patterns (Carobbio et al., 2020).

To expedite advancements in survival prediction research, researchers are harnessing the capabilities of AI algorithms by utilizing extensive survival-related data from public databases such as the Cancer Genome Atlas Program (TCGA) (Tomczak et al., 2015), and NCI Genomic Data Commons (GDC) (Jensen et al., 2017; Shen et al., 2019; Malik et al., 2021; Mirbabaie et al., 2021; Arjmand et al., 2022; Fan et al., 2023; Pellegrini, 2023). In addition, the diversity and heterogeneity of diseases hinder the development of a universally applicable survival prediction pipeline (Kourou et al., 2015; Hao et al., 2023).

**Figure 1:**
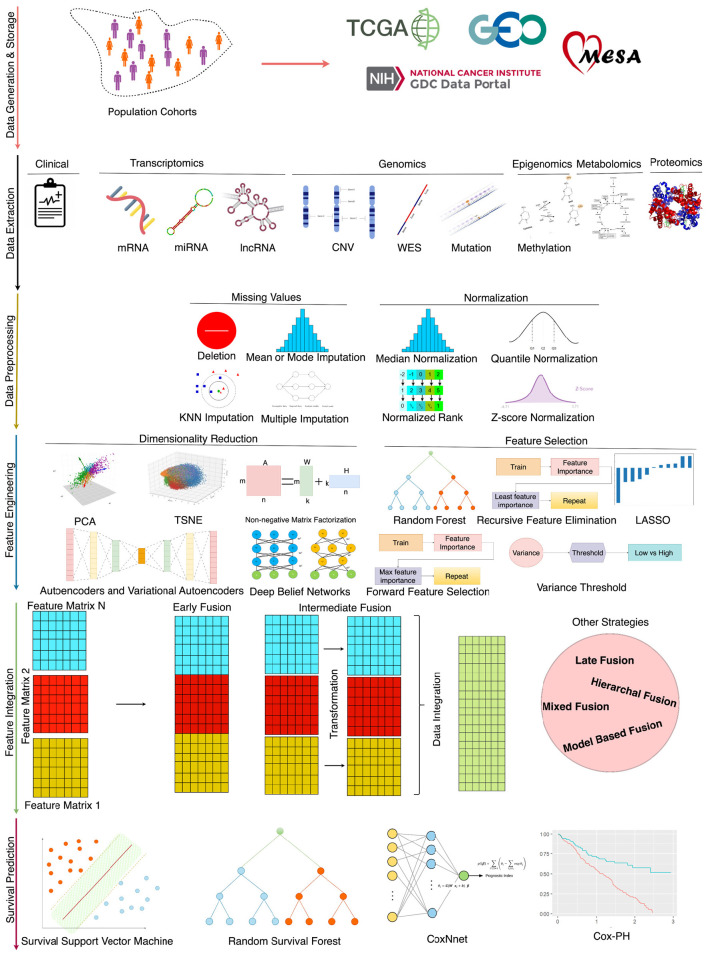
An end-to-end survival prediction pipeline.

Driven by the necessity for disease-specific predictors, there is a concerted effort to develop more accurate and powerful predictive tools (Baek and Lee, 2020; Jiang et al., 2020; Benkirane et al., 2023). Figure 1 illustrates that for the advancement of survival predictors, public databases provide a spectrum of clinical data (Jung et al., 2023; Qian et al., 2023) and encompass nine diverse omics data modalities, including gene expression (mRNA), micro RNA (miRNA), DNA methylation, copy number variation (CNV), long non-coding RNA (lncRNA), proteomics, metabolic, whole exome sequencing (WES) and mutation (Baek and Lee, 2020; Malik et al., 2021; Han et al., 2022; Jiang et al., 2022). In each data modality, there exists an array of missing values that hinder survival predictors learning. Extensive research is being conducted to impute missing values by using different techniques such as deletion, multiple, K-nearest neighbor (KNN), and median imputation (Van Buuren et al., 1999; Garćıa-Laencina et al., 2015; Chai et al., 2021b). In addition, various normalization methods are also being used to normalize feature space such as quantile (Zhao et al., 2020), variance threshold (Bolstad et al., 2003), and rank normalizations (Ni and Qin, 2021).

In the development of survival prediction pipelines, researchers are trying to unlock the potential of various data modalities by assessing predictor performance with individual modalities and combinations of multiple data modalities across diverse types of diseases (Lee et al., 2020; Hao et al., 2023; Pellegrini, 2023). When data from different modalities is combined, survival predictors' input feature space becomes very large which impedes the performance of AI approaches (Feldner-Busztin et al., 2023). Researchers are trying to explore feature engineering approaches such as random forest importance (RFI), and recursive feature elimination (RFI) (Wang et al., 2022), principal component analysis (PCA) (Lv et al., 2020; Jiang et al., 2022), non-negative matrix factorization (NMF) (Tang et al., 2021), and autoencoders (AEs) (Li et al., 2020; Wang et al., 2020; Owens et al., 2021). Moreover, in an end-to-end survival predictive pipeline, apart from the selection of appropriate data and feature engineering strategy, designing appropriate survival prediction models is also an active area of research (Deepa and Gunavathi, 2022).

Under different aforementioned directions, the recent 3 years have witnessed around 74 different survival predictors for different diseases. To further accelerate and expedite the development of more powerful predictors, in the last 10 years, from time to time, researchers have published 22 different review articles. These articles primarily aim to summarize the latest trends and developments in data modalities, feature engineering methods, and AI models specifically related to survival prediction. However, the focus of these reviews is often constrained to either a singular disease or multiple subtypes of cancer, highlighting a limited scope within the broader landscape of survival prediction research (Herrmann et al., 2021; Pobar et al., 2021; Boshier et al., 2022; Deepa and Gunavathi, 2022; Feldner-Busztin et al., 2023; Rahimi et al., 2023). More comprehensive details about the scope of existing review articles in terms of contributions and drawbacks are summarized in Table 2 and Section 3. Following the need for a comprehensive review article for survival prediction, the contributions of this paper are manifold:

- It consolidates a diverse array of 22 survival prediction review papers, bringing together their scopes and limitations under a unified umbrella. This compilation serves as a valuable resource for researchers seeking high-level insights and pertinent information in the field.
- It provides comprehensive insights into 74 survival prediction articles published between 2020 and 2023.

The objective is to delve into diverse aspects of the field, extract and furnish useful information from these articles under the following different research questions (RQs) and objectives: (i) What is the distribution of 74 research articles across 44 different diseases, and how does it vary among cancer subtypes and other diseases? (ii) How do studies address the spectrum of survival prediction, from a broader perspective covering multiple cancer subtypes to individual subtypes? (iii) What are the predominant survival endpoints used in studies, and how are studies distributed across four endpoints overall survival (OS), disease-free survival (DFS), progression-free survival (PFS), and biochemical recurrence (BC)? (iv) What are the most commonly used public and private data sources in existing survival prediction studies and the types of data they encompass? (v) What are the most commonly used omics data modalities and their associations with different diseases and survival endpoints? (vi) Which clinical features are most commonly employed in survival prediction studies? (vii) How have feature engineering techniques evolved across different data modalities, diseases, and survival endpoints in survival prediction studies? (viii) Which specific statistical, machine learning (ML), and deep learning (DL) survival prediction algorithms have been applied to diverse diseases and survival endpoints? (ix) Which survival prediction studies have made their source codes publicly available, and what types of methods are available in open-source survival prediction frameworks? (x) What are the most commonly utilized survival prediction evaluation measures? (xi) Which conferences and journals predominantly publish survival prediction studies?

## 2 Background

Survival prediction makes use of patient-specific molecular information and clinical signatures to forecast a wide range of events at particular time intervals (Pellegrini, 2023). The most common events include recurrence, metastasis, response, hospitalization, recovery, and progression of a disease. Some of these events represent similar contexts, i.e., metastasis and progression, both contribute to the overall progression of the condition/cancer (Murthy et al., 2004). Survival prediction events are generally categorized into 4 different survival endpoints namely, overall survival (OS) (Driscoll and Rixe, 2009), disease-free survival (DFS) (Sargent et al., 2005), progression-free survival (PFS) (Gyawali et al., 2022), and biochemical recurrence (BC) (Boorjian et al., 2011). Survival endpoints serve as crucial measures for assessing the outcomes of interventions, indicating the duration until specific events occur. Therefore, events are essentially the occurrences that contribute to the survival endpoints (Fiteni et al., 2014). These endpoints are critical to examine the trajectory of a particular disease (Fiteni et al., 2014; Gyawali et al., 2022). These survival endpoints are clearly defined in Table 1.

**Table 1:** Common clinical/survival endpoints in cancer studies.

**Endpoint**	**Definition**	**Example in cancer**
Overall survival (OS)	Time from diagnosis or treatment initiation to death from any cause	Time from breast cancer diagnosis to death from any cause
Disease-free survival (DFS)	Time from treatment initiation to disease recurrence or death from any cause, whichever occurs first	Time from colorectal cancer surgery to disease recurrence or death
Progression-free survival (PFS)	Time from treatment initiation to disease progression or death from any cause, whichever occurs first	Time from lung cancer treatment to tumor progression or death
Biochemical recurrence (BC)	Return of cancer based on biochemical markers, typically measured in prostate-specific antigen (PSA) levels	Increase in PSA levels after prostate cancer treatment

Survival prediction is time to event approach with two distinct aspects, i.e., survival and hazard function (Kleinbaum and Klein, 1996). Survival function describes the probability that a subject survives longer than some specified time *t*. Mathematically, it is expressed as:


$$\begin{array}{l} S\left(t\right)=P\left(T>t\right), \end{array}$$

where *T* is the random variable for survival time, *t* is a specific value of interest for *T*. For instance, *S*(10) represents the probability of survival beyond 10 years without experiencing a specific event. As time passes, *S*(*t*) decreases, reflecting the reduction in the probability of surviving without the occurrence of event *E* up to time *t*.

In comparison, the hazard function illustrates the probability of an event *E* occurring at a specific time interval (Δ*t*) with a prior assumption that the event has not taken place. The probability that the event *E* occurs within a very small time interval Δ*t* around time *t* is given by the conditional probability:


$$\begin{array}{l} P\left(t\leq T<t+\Delta t|T\geq t\right) \end{array}$$

Dividing this probability by the length of the time interval (Δt) gives the rate of occurrence of the event at time *t*. The limit as the time interval (Δt) approaches zero gives the instantaneous rate of occurrence at time *t*. Mathematically, this is represented as:


$$\begin{array}{l} h(t)=\underset{\left(\Delta t\to 0\right)}{\lim}\frac{Pr(t\leq T<t+\Delta t|T\geq t)}{\Delta t}\\ \;=\frac{f(t)}{S(t)} \end{array}$$

$$\begin{array}{r} Pr(t\leq T<t+\Delta t|T\geq t)=\\ P(\text{individual fails in interval}|[t,t+\Delta t]\\ \text{survivaluptotimet}) \end{array}$$

where *f*(*t*) represents the probability density function of survival time. Thus, survival function *S*(*t*) shows that the subject survives beyond a specific time point and hazard function h(t) complements this by providing a risk rate that a patient does not survive in a specific time interval conditioned on having survived thus far. Moreover, *S*(*t*) is always monotonic in nature, however *h*(*t*) is classically assumed to follow increasing Weibull, decreasing Weibull, or lognormal survival curves (Kleinbaum and Klein, 1996; Murthy et al., 2004).

## 3 A look-back into existing review studies

In recent years multiple review papers have been published and the objective of each review revolves around summarizing fundamental concepts in survival prediction and identifying trends in statistical, ML, and DL algorithms that have been utilized in the development of survival predictors. Table 2 illustrates a high-level overview of the existing 22 review articles in terms of their review scope and limitations. This comprehensive summary aims to assist researchers in locating specific information within relevant articles more effectively.

**Table 2:** The scope and limitations of current survey papers.

**References**	**Citations**	**Number of articles covered**	**Scope**	**Shortcomings**
Deepa and Gunavathi (2022)	17	37	Cancer survival, subtypes, and recurrence prediction across seven cancer subtypes i.e., breast, lung, gastric, cervical, oral, cystic fibrosis, and multi- cancers	Does not take into account all types of cancers, and other diseases on the basis of multiomics and clinical data.
Herrmann et al. (2021)	59	NS	A benchmark of different ML and statistical survival analysis methods on multiple cancer datasets from TCGA i.e., bladder, breast invasive, colon, esophegeal, head-neck squamous, kidney renal, cervical kidney, acute myloid leukemia, low grade glioma, liver hepatocellular, lung adenocarcinoma, lung squamous, ovarian cancer, oancreatic, sarcoma, skin cutaneous, stomach, and Uterine corpus cancers.	A benchmark with a limited number of methods for survival modeling.
Rahimi et al. (2023)	2	13	Cervical cancer (CC) survival analysis based on ML and statistical methods to predict Disease-free survival (DFS), progression-free survival (PFS), and overall survival (OS)	The review is confined to cervical cancer survival prediction and does not encompass deep learning-based methods.
Bashiri et al. (2017)	80	17	Survival Prediction based on gene expression data across Mantle cell lymphoma, esophageal adenocarcinoma, Esophageal squamous cell carcinoma, Non- small cell lung carcinomas, Diffuse large B-Cell lymphoma (DLBCL), astrocytic tumor, and Lung cancer	Multiomics data is not extensively discussed, as it is understood that the emphasis on gene expression alone may not define the survival of a subject.
Tewarie et al. (2021)	23	27	Continous and discrete-time survival prediction across glioblastoma based on magnetic resonance images (MRI), genomics, and clinical data	The review paper does not include a discussion of survival prediction models. Also, the role of multiomics data in survival prediction has not been explored.
Westerlund et al. (2021)	23	NS	Risk prediction in cardiovascular diseases (CVD) based on clinical, and image data, and molecular signatures such as single nucleotide polymorphism (SNP).	-
Kresoja et al. (2023)	5	NS	An overall spectrum of survival prediction in cardiovascular diseases is presented based on the image, omics, and clinical data.	-
Kaur et al. (2022)	33	62	The authors reviewed how data mining and ML are transforming medical decision-making, focusing on cancer survival research. They analyzed 62 articles from the past 15 years, noting a shift from traditional methods to ML and DL, due to the availability of large digital datasets. The study highlights a move toward using clinical data with smaller sample sizes and an increasing use of DL and hybrid approaches. They identify ten open research issues and suggest future research directions to improve cancer survival predictions, offering insights for both new and experienced researchers.	Limited papers cohort and lack of discussion on multi-omics data modalities and survival endpoints.
Wiegrebe et al. (2023)	4	58	Survival prediction with DL models from five major categories i.e., discrete-time, piece- wise exponential, parametric, ranking-based, and ordinary differential equation (ODE)	While it encompasses numerous models, the paper still lacks coverage of information related to ML models.
Salerno and Li (2023)	5	NS	ML and DL based methods are discussed for survival analysis with a focus on high dimensionality of the data. Mainly, regularized cox models, support vector machines, random survival forests, boosting, and artificial neural networks are presented.	Only a handful of methods are discussed in this specific review whereas, the number of methods used to deal with high dimensional data is significant in number.
Pobar et al. (2021)	15	16	DL for survival prediction in palliative cancer patients (advanced cancer patients) on the basis of radiomics data and evaluation based on Palliative Prognostic Score (PaP), Palliative Prognostic Index (PPI) and Number of Risk Factors (NRF)	The prime focus is only related to radiomics-based methods.
Bakasa and Viriri (2021)	16	NS	Pancreatic survival prediction models and the use of DL models such as image segmentation, and feature extraction. Different concepts like image segmentation and feature extraction are discussed in detail with less emphasis on their utilization in ML or DL-based survival prediction. In addition, very few studies are referred related to pancreatic cancer survival prediction.	
Ahmed (2005)	252	NS	The internal components of artificial neural networks (ANNs)	Authors provide a rough overview of artificial neural networks (ANNs). At the time of this publication, there was approximately very little attention given to survival prediction using ML and DL-based models. Therefore, the review discusses only the internal workings of ANNs rather than discussing the details of survival prediction and the role of AI in it.
Kantidakis et al. (2022)	3	24	Studies related to survival prediction are presented in two different settings i.e., setting 1: time is added as part of the input features and a single output node is specified, setting 2: multiple output nodes are defined for each time interval	Authors discuss different types of neural network setting used for survival analysis yet they did not categorize all the studies related to survival analysis on the basis of the type of neural network setting being used.
Altuhaifa et al. (2023)	0	30	Studies related to cancer survival prediction. The authors present databases utilized for the prediction of cancer survival prediction along with feature selection algorithms, types and nature of features, survival prediction models, and limitations.	Lack of characterization with respect to the multiomics-based data modalities.
Wekesa and Kimwele (2023)	0	NS	radiomics, and multiomics studies related to different factors that play a critical role in various diseases i.e., miRNA, circRNA, and so on are presented. The prime focus is on data integration techniques based on DL for interaction prediction, disease diagnosis, and treatment.	Only a handful of studies are covered
Kvamme and Borgan (2021)	47	NS	Authors discuss in detail the architectures and schemes utilized to predict survival in a discrete or continuous fashion.	-
Feldner-Busztin et al. (2023)	13	NS	Dimensionality reduction in ML models with context to cancer subtype identification, and survival prediction.	The prime focus is on the use of dimensionality reduction in multiomics related tasks. The role of dimensionality reduction in survival prediction has not been covered in this review.
Boshier et al. (2022)	0	17	Survival prediction in esophageal adenocarcinoma is discussed on the basis of clinical data. In addition, various survival prediction models are evaluated on new validation data comprised of 2,450 patients.	Only limited to a single cancer and the focus is only related to clinical data.
Gupta et al. (2018)	38	16	Prognostication in terms of esophageal and gastroesophageal junction cancer on the basis of image and clinical data.	Lack of multiomics-based analysis.
Wissel et al. (2022)	-	-	Authors discuss and propose new standardized benchmark datasets and their splits for survival prediction, obtained from TCGA, TARGET, and ICGC databases. The comparison of the AI-based and statistical models is also presented in the paper which shows that statistical models often beat AI-based models in time to event prediction with multiomics data.	-
Lee and Lim (2019)	62	NS	Different concepts related to survival analysis are discussed i.e., survival functions, Kalpan Meier estimators, and log-rank test. In addition, multiple time-to-event modeling approaches are also presented in detail such as, Cox-PH model, random survival forest, survival support vector machines, bagging, cox boosting, and artificial neural networks.	Limited coverage of omics-based modalities and an in-depth discussion.
Guan et al. (2022)	40	NS	Subtyping and risk prediction in Schizophrenia.	-
Mo et al. (2022)	0	NS	A comparison of 12 supervised ML models to predict the outcome of head and squamous cell carcinoma i.e., bayesian network, naive Bayes, logistic regression, generalized linear model, k-nearest neighbor, decision tree, random forest, bootstrap aggregating, and AdaBoost, gradient boosting trees, neural network, and support vector machine. In addition, important genes are further validated using a variety of wet lab experiments.	Only a single multiomics data is used for the comparison of different survival outcome prediction models, whereas multiple datasets can show the generalizability of the models on the data belonging to various demographic locations.
Ours	-	74	A systematic analysis of diverse survival prediction literature. This review encompasses ML, DL, and statistical survival predictors across more than 30 different diseases. In addition, the review addresses diverse research questions related to the distribution of survival predictors, databases, data modalities, feature engineering methods, survival prediction models, source codes and libraries for the development of survival predictors, and various evaluation measures.	This review paper focuses solely on current trends in survival prediction, omitting basic terminologies and mathematical formulations. For a concise mathematical overview, readers are advised to consult earlier review papers (Lee and Lim, 2019; Kvamme and Borgan, 2021).

In Table 2, a comprehensive analysis of the scope of review articles indicates that existing studies can be classified into three distinct groups. (I) Nine review papers primarily focus on the application of DL algorithms in survival prediction (Ahmed, 2005; Bakasa and Viriri, 2021; Kvamme and Borgan, 2021; Pobar et al., 2021; Kantidakis et al., 2022; Altuhaifa et al., 2023; Salerno and Li, 2023; Wekesa and Kimwele, 2023; Wiegrebe et al., 2023), (II) seven review papers summarize the application of ML algorithms in survival prediction (Gupta et al., 2018; Lee and Lim, 2019; Boshier et al., 2022; Guan et al., 2022; Mo et al., 2022; Wissel et al., 2022; Feldner-Busztin et al., 2023), andsix review papers summarize survival prediction methods from three different categories namely statistical, ML, and DL methods (Bashiri et al., 2017; Herrmann et al., 2021; Tewarie et al., 2021; Westerlund et al., 2021; Deepa and Gunavathi, 2022; Rahimi et al., 2023).

On the other hand, in the realm of disease specific survival predictors scope of existing review papers is limited. For instance, eight papers only summarize survival predictors on single disease or subtype of cancer, i.e., cervical cancer (Rahimi et al., 2023), glioblastoma (Tewarie et al., 2021), esophageal adenocarcinoma (Boshier et al., 2022), esophageal and gastroesophageal junction cancer (Gupta et al., 2018), head and squamous cell carcinoma (Mo et al., 2022), palliative cancer patients (Pobar et al., 2021), cardiovascular diseases (CVD) (Westerlund et al., 2021; Kresoja et al., 2023), and schizophrenia (Guan et al., 2022). Although four papers cover multiple subtypes of cancer but they cover only handful of eight different subtypes such as, breast, lung, gastric, colon, esophageal, ovarian cancers and so on.

While the scope of survival prediction extends beyond multiple diseases, existing review papers fall short to summarize current trends of data modalities, feature engineering approaches and survival prediction models. For example, Deepa and Gunavathi (2022) specifically address the primary categories of data modalities used for survival prediction, namely multiomics and clinical data. However, the review does not extensively explore trends and patterns related to the nine different omics types i.e., gene expression (mRNA), micro RNA (miRNA), methylation, copy number variation (CNV), whole exome sequencing (WES), long noncoding RNA (lncRNA), mutation, metabolic, and proteomics, or clinical features associated with distinct cancer subtypes. Similarly, Westerlund et al. (2021) do not explore the potential of multiomics data in terms of cardiovascular diseases. In addition, various review papers completely neglect to address feature engineering in survival prediction (Ahmed, 2005; Bashiri et al., 2017; Gupta et al., 2018; Pobar et al., 2021; Kantidakis et al., 2022; Rahimi et al., 2023). For instance, Feldner-Busztin et al. (2023) despite their focus on dimensionality reduction, fall short in providing a comprehensive summary of current trends in feature engineering approaches with respect to diseases and data modalities. Furthermore, a small portion of these review papers cover details of few state of the art survival prediction models (Ahmed, 2005; Kantidakis et al., 2022; Wiegrebe et al., 2023). While current review papers summarize survival prediction pipelines partially, there is a necessity to bring diverse information into a unified platform which offers comprehensive insights into patterns and trends associated with survival prediction pipelines.

## 4 Methodology

**Figure 2:**
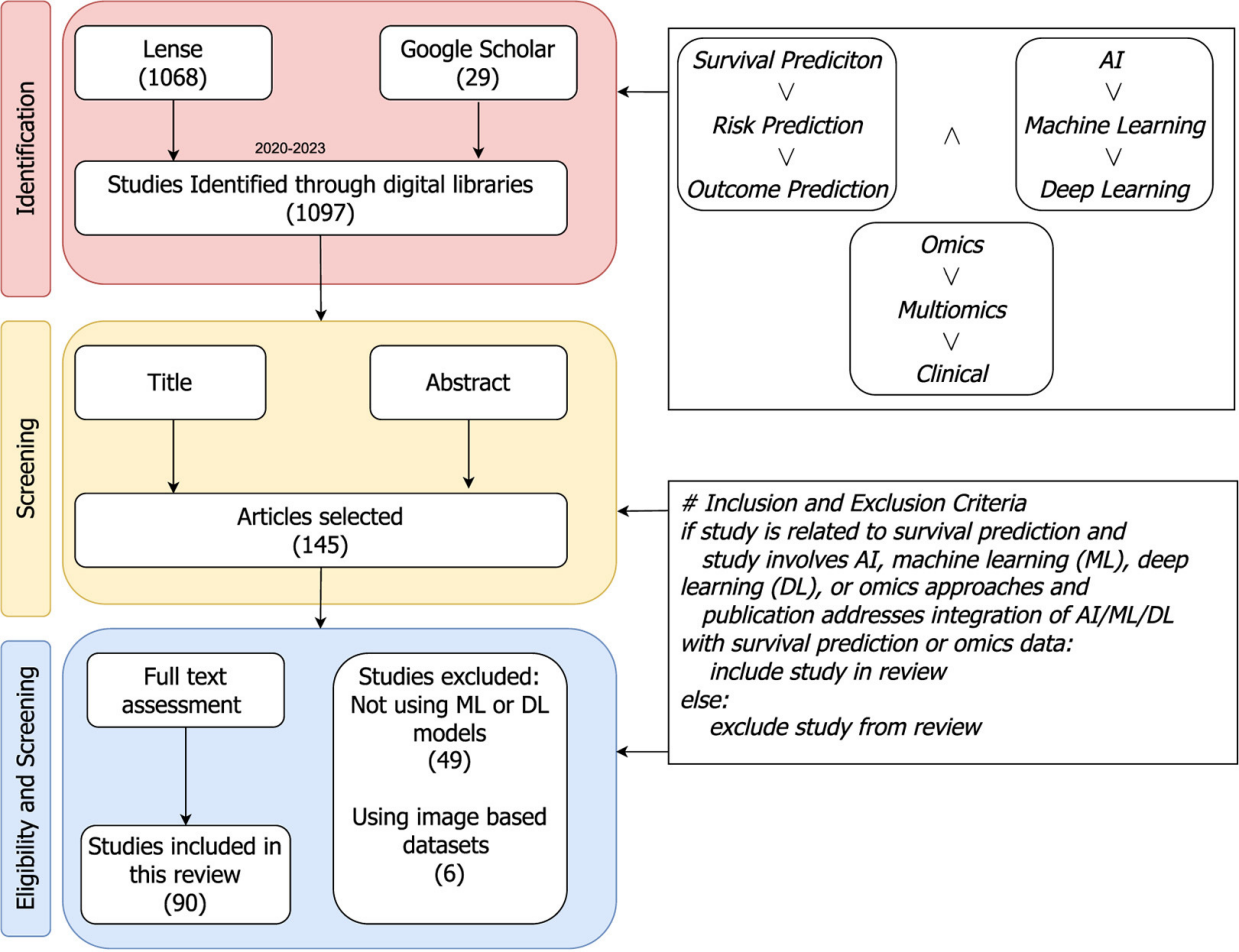
PRISMA flow diagram: a step-by-step process for articles search and their inclusion or exclusion criteria to generate a set of studies for further in-depth trends analysis. The included papers are collected from Jan 2020 to Jul 2023.

This section explains different steps or stages of preferred reporting items for systematic review and meta-analyses (PRISMA) strategy (Moher et al., 2010), which is used to gather relevant papers on survival analysis. Figure 2 provides a visual representation of various stages form PRISMA that are summarized in the following subsections.

### 4.1 Search strategy

In Figure 2, the identification stage illustrates combinations of different keywords that are used to search research articles. The keywords block has two different types of operators “∧” and “∨” operators. On the basis of these operators one keyword from each block is selected and various search queries are formulated such as, “*SURVIVAL PREDICTION AND AI AND OMICS”*, “*SURVIVAL PREDICTION AND AI AND Multiomics”*, “*SURVIVAL Machine Learning AND OMICS”*, and so on. These queries are utilized in literature search engines like lens (https://www.lens.org/), and Google Scholar for literature search from Jan 2020 to Jul 2023.

### 4.2 Eligibility and screening strategy

With an aim to retain literature related to survival prediction, two different screenings are performed on the basis of the following criteria;

- Articles that use only image-based datasets for survival prediction.
- Articles that do not make use of ML, DL, or statistical methods for survival prediction.
- Articles with closed access.

Initially, guided by the title and abstract of the articles, more than 800 studies are discarded. Subsequently, at the final step, based on a comprehensive review of the full text a second screening is performed, resulting in the exclusion of an additional 20 studies. Ultimately, 90 papers are selected for the final comparison and discussion of survival prediction.

## 5 Results

### 5.1 RQ I, II, III: survival predictors distribution analysis across diseases and survival endpoints

The primary aim of this section is to summarize the distribution of survival predictors across various diseases and survival endpoints. Predictors distribution analysis under individual diseases offers insights into the most active trends of predictors associated with specific diseases. This consolidated distribution provides a centralized platform to access valuable information about their disease of interest. Similarly, examining the distribution of articles across survival endpoints is valuable for identifying current trends in forecasting multiple events. This approach not only enhances our understanding of the current state of predictive modeling but also facilitates researchers in efficiently accessing information specific to their desired endpoints. Through this exploration, we aim to contribute to a deeper understanding of the diverse landscape of survival prediction research and its applications across various diseases and endpoints.

Table 3 illustrates disease specific predictors distribution for both cancer and other diseases, respectively. In the last 3 years, 74 predictors have been designed for different cancer subtypes related survival prediction (Tan et al., 2020; Fan et al., 2023; Majji et al., 2023) while only 17 predictors have been designed for other diseases such as cardiovascular diseases, COVID-19, cardiomyopathy, esophagectomy and trauma (Kantidakis et al., 2020; Abdelhamid et al., 2022; Feng et al., 2022; Farahani et al., 2023; Qian et al., 2023; Rahman et al., 2023).

**Table 3:** Distribution of survival predictors across individual diseases.

**Disease subtype**	**Number of studies**	**References**
Nasopharyngeal carcinoma:	1	Miao et al., 2022
HER2-negative metastatic breast cancer	1	Wang J. et al., 2023
Tripple negative breast cancer	1	Zhang et al., 2023
Breast invasive carcinoma	1	Hao et al., 2023
Colon adenocarcinoma	1	Lv et al., 2020
Gastric cancer	1	Li et al., 2020
Gastrointestinal cancer	1	Jung et al., 2023
Adult diffuse glioma	1	Yang Q. et al., 2020
Invasive ductal carcinoma	1	Lin et al., 2022
Pancreatic cancer undergoing biliary drainage	1	Zhou et al., 2023
Kidney renal clear cell carcinoma	2	Zhao L. et al., 2021; Hao et al., 2023
Lung squamous cell carcinoma	1	Hao et al., 2023
Cervical cancer	1	Hu Q. et al., 2021
Neuroblastoma	1	Wang et al., 2022
Rectal cancer	1	Zhang J. Z. et al., 2022
Colon cancer	3	Tong D. et al., 2020; Yang H. et al., 2020; Lee et al., 2023
Liver cancer	1	Wang et al., 2020
Esophageal carcinoma	2	Yu et al., 2020; Bichindaritz and Liu, 2022
Stomach adenocarcinoma	1	Zhao L. et al., 2021
Ovarian serous cystadenocarcinoma	2	Zhao L. et al., 2021; Bichindaritz and Liu, 2022
Kidney renal clear cell carcinoma	2	Zhao L. et al., 2021; Jiang et al., 2022
Lower grade glioma	1	Wu et al., 2022
Head-and-neck squamous cell carcinoma	1	Zhao L. et al., 2021
Bladder cancer	3	Chai et al., 2021a; Tang et al., 2021; Chauhan et al., 2023
Bladder urothelial carcinoma	1	Zhao L. et al., 2021
Renal cell carcinoma		Tong et al., 2021; Shetty et al., 2023
Lymphoma	1	Li et al., 2023
Hepatocellular carcinoma	3	Owens et al., 2021; Zhang R. et al., 2022; Wang X. et al., 2023
Ovarian cancer	5	Hira et al., 2021; Pawar et al., 2022; Wu and Fang, 2022; Lang et al., 2023; Wang X. et al., 2023
Glioblastoma	4	Du et al., 2020; Kazerooni et al., 2021; Redekar et al., 2022; Wu and Fang, 2022
Prostate cancer	3	Doja et al., 2020; Li et al., 2021; Pellegrini, 2023
Non-small cell lung cancer	5	Zhang Z.-S. et al., 2021; Ellen et al., 2023; Manganaro et al., 2023; Wang Q. et al., 2023
Pancreatic cancer	3	Baek and Lee, 2020; Han et al., 2022
Breast cancer	10	Tong L. et al., 2020; Hu S. et al., 2021; Malik et al., 2021; Zhou et al., 2021; Wu and Fang, 2022; Zhang J. Z. et al., 2022; Othman et al., 2023; Palmal et al., 2023; Zarean Shahraki et al., 2023; Zhu et al., 2023
Lung adenocarcinoma	4	Jiang et al., 2020; Lee et al., 2020; Zhang S. et al., 2022; Bhat and Hashmy, 2023
Pan-cancer	7	Tan et al., 2020; Zhang X. et al., 2021; Zheng et al., 2021; Yin et al., 2022; Benkirane et al., 2023; Fan et al., 2023; Majji et al., 2023
Colorectal cancer	1	Willems et al., 2023
Atherosclerosis	3	Hathaway Q. A. et al., 2021; Hathaway Q. et al., 2021; Qian et al., 2023
Myocardial infarction	1	Feng et al., 2022
Stroke	1	Feng et al., 2022
COVID-19	1	Richard et al., 2022
Cardiovascular disease	6	Unterhuber et al., 2021; Vahabi et al., 2021; Xu et al., 2021; Zeng et al., 2021; Feng et al., 2022; Moreno-Sanchez, 2023
Liver transplant	2	Kantidakis et al., 2020; Raju and Sathyalakshmi, 2023
Trauma	1	Abdelhamid et al., 2022
Metastatic urothelial cancer	1	Tarango et al., 2023
Hypertrophic cardiomayopathy	1	Farahani et al., 2023
Papillary thyroid carcinoma	1	Lun et al., 2024
Esophagectomy	1	Rahman et al., 2023
Diffuse large B-cell lymphoma	1	Pant et al., 2023

**Figure 3:**
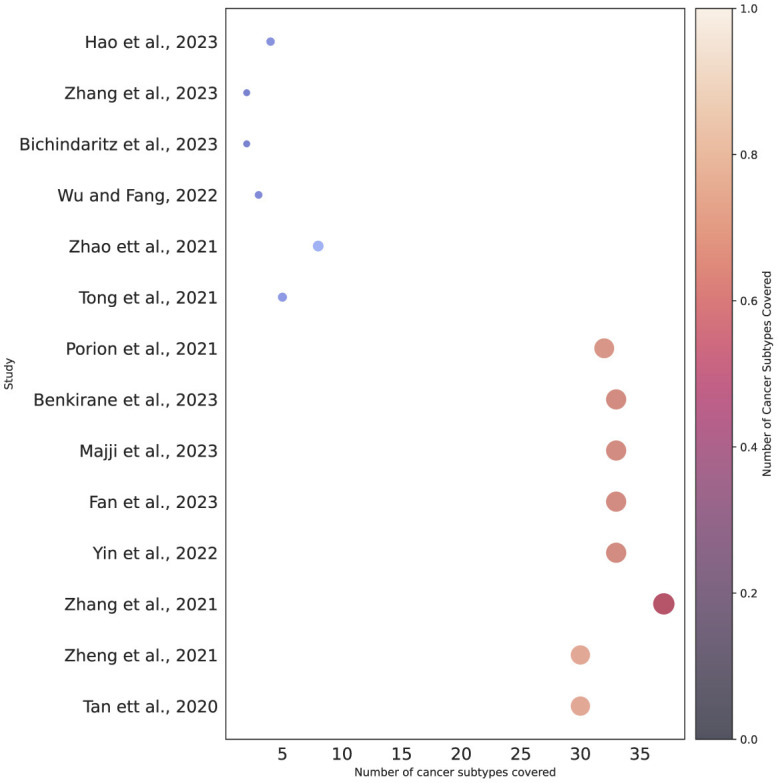
Cancer subtypes coverage based on pan-cancer or individual subtype settings.

To date, approximately more than 100 different cancer subtypes have been identified (Grever et al., 1992). However, a deeper analysis of the last 3 years reveals that survival prediction models have been developed for only 40 distinct cancer subtypes, as outlined in Table 3. Among 36 different subtypes, most of the predictors have been designed for breast cancer, lung adenocarcinoma, ovarian cancer, and glioblastoma. On the other hand, seven different predictors have been designed for pancancer. Notably, there is a difference between other cancer types and pan-cancer because under this paradigm predictors simultaneously deal with multiple cancer subtypes. Pan-cancer based survival prediction entails predicting patient survival outcomes using data and models applicable to various cancer types (Fan et al., 2023). Instead of focusing on just one type of cancer, this approach draws on data from multiple cancers to identify shared patterns and markers that influence survival. By combining a diverse array of genetic, molecular, and clinical features that are common across different cancers, this method aims to improve the accuracy of survival predictions (Wu et al., 2023). For the development of pan-cancer based predictors, there exists public data having more than 30 distinct cancer subtypes (Liu et al., 2018). However, researchers are utilizing different subsets for the development of predictors (Fan et al., 2023). Figure 3 provides an overview of multiple survival prediction studies that encompass a range of cancer subtypes, either within a pan-cancer context or within the context of predicting survival for different subtypes. A total of 14 studies have taken into account multiple cancer subtypes whereas the majority of the studies have only covered only a single type of cancer subtype such as colorectal cancer (Willems et al., 2023), lymphoma (Li et al., 2023), colon adenocarcinoma (Lv et al., 2020), gastric cancer (Li et al., 2020), and so on.

**Figure 4:**
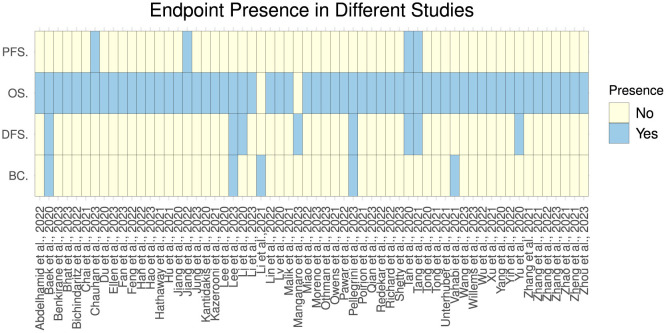
Survival endpoint distribution across diverse studies.

**Figure 5:**
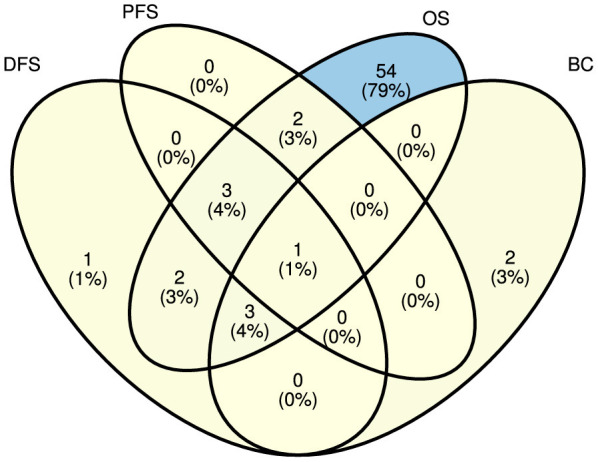
Distribution of explored survival prediction streams from existing literature. DFS, disease-free survival; PFS, progression-free survival; OS, overall survival; BC, biochemical recurrence.

Figures 4, 5 illustrate predictors distribution across survival endpoints. A majority of studies 67 (76%) have OS as an endpoint of survival prediction (Chai et al., 2021a; Abdelhamid et al., 2022; Benkirane et al., 2023; Bhat and Hashmy, 2023), whereas eight studies have incorporated multiple survival endpoints in their analysis. Out of eight studies, three studies have incorporated DFS and BC (Lee and Wang, 2003; Baek and Lee, 2020; Pellegrini, 2023). Two studies have incorporated OS, DFS, and PFS (Tan et al., 2020; Tang et al., 2021) and two studies have OS, and PFS as the survival endpoints (Jiang et al., 2022; Chauhan et al., 2023), one focuses on OS and DFS (Pant et al., 2023). A single study has focused on DFS only (Manganaro et al., 2023), and two only on BC (Li et al., 2021; Vahabi et al., 2021). The rest of studies either did not explicitly specify their endpoints for survival prediction or predominantly concentrated on predicting patients' survival outcomes without a specific focus on distinct survival endpoints.

### 5.2 RQ IV: survival prediction data availability in public and private sources and opportunities for development of predictors

Survival prediction models development relies on the quality and quantity of annotated data, which is generated through extensive wet lab experiments. Experimental findings are stored in different types of databases that open new doors for the development of survival prediction applications. However, there exist multiple databases and each database encompasses particular diseases and modality specific survival data. For instance, CGGA (Zhao Z. et al., 2021) focuses on brain tumors, and MESA (Bild et al., 2002) contains data related to atherosclerosis. To accelerate the development of more competent survival predictors, it is essential to summarize which database contains which type of disease and what data modalities. In the highlight of research question IV, Table 4 illustrates public databases details in terms of diseases and data modalities they offer.

**Table 4:** The ample collection of survival data within diverse public databases.

**Data source**	**Diseases covered**	**Types of data**	**URL**	**Description**
GDAC Broad Firehose (Voet et al., 2022)	38 different cancer subtypes	Clinical, CNV, methylation, miRNA, mRNA, mutation, proteomics	https://gdac.broadinstitute.org/	The Firehose platform provides processed and analyzed data from TCGA, making it accessible to researchers for further analysis and interpretation.
Chinese Glioma Genome Atlas (CGGA) (Zhao Z. et al., 2021)	Brain Tumor	Clinical, single cell RNA, mRNA, image, and microarray	http://www.cgga.org.cn/	The Chinese Glioma Genome Atlas (CGGA) is a genomic database focused on glioma, providing comprehensive molecular characterization and clinical information to advance the understanding and treatment of glioma tumors.
TARGET	Pediatric cancers such as osteosarcoma, neuroblastoma, rhabdoid cancer, Wilms, acute myloid leukemia, acute lymphoblastic leukemia	Clinical, mRNA, miRNA, methylation, proteomic and CNV	https://www.cancer.gov/ccg/research/genome-sequencing/target	The TARGET (Therapeutically Applicable Research to Generate Effective Treatments) NCI (National Cancer Institute) database is dedicated to pediatric cancers, offering molecular and clinical data to facilitate research and the development of targeted therapies for pediatric cancer patients.
SEQC (Zhang et al., 2015)	Neuroblastoma	Microarray, and mRNA	–	RNA-seq and microarray data to predict clinical/survival endpoints for neuroblastoma,
MsigDb (Liberzon et al., 2011)	–	Curated gene sets, motif gene sets, gene ontology terms, oncogenic signatures, and immunologic signatures	https://www.gsea-msigdb.org/gsea/msigdb	The Molecular Signatures Database (MSigDB) is a collection of annotated gene sets, pivotal for gene set enrichment analysis, encompassing diverse biological pathways and functions, aiding researchers in studying gene expression patterns.
GTEX (Stanfill and Cao, 2021)	54 non-diseased tissue sites across nearly 1,000 individuals	Single cell, mRNA, methylation, chip-seq, histology images	https://www.gtexportal.org/home/	The Genotype-Tissue Expression (GTEx) project is a comprehensive research initiative that characterizes the genetic and tissue-specific gene expression patterns across a diverse set of human tissues, providing valuable insights into the relationship between genetic variation and gene regulation.
Kaggle	Not disease-specific	Clinical	https://www.kaggle.com/	Kaggle is an online platform that hosts data science competitions and facilitates collaboration among data scientists, offering datasets and a community for learning and problem-solving. In addition, Kaggle is not specifically designed for omics-based datasets or studies.
MESA (Bild et al., 2002)	Heart diseases	Clinical, genetic, and imaging	https://www.mesa-nhlbi.org/default.aspx	The Multi-Ethnic Study of Atherosclerosis (MESA) MESA is a research study by the National Heart, Lung, and Blood Institute, involves 6,000+ individuals from six U.S. communities, assessed at affiliated university clinics
UCSC Xena (Goldman et al., 2018)	Various cancer subtypes that are present in TCGA	Clinical and omics data modalities associated with TCGA	https://xena.ucsc.edu/	UCSC Xena is a bioinformatics platform offering a user-friendly interface for the exploration and visualization of integrated multi-omic and clinical datasets, enabling researchers to analyze and interpret diverse biological and disease-related information collaboratively.
GEO (Clough and Barrett, 2016)	Plethora of diseases such as cardiovascular and neurological diseases and cancers	Clinical, mRNA, miRNA, CNV, methylation, chromatin interaction, DNA modification, splicing, lncRNA, and mutation	https://www.ncbi.nlm.nih.gov/geo/	The Gene Expression Omnibus (GEO) is a publicly accessible repository, maintained by the National Center for Biotechnology Information (NCBI), housing a diverse collection of high-throughput functional genomics datasets, enabling researchers to freely access and analyze gene expression and other omics data across a broad spectrum of biological conditions and diseases. GEO does not have a specific or dedicated portal for survival prediction datasets.
TCGA-GDC (Tomczak et al., 2015)	More than 40 cancer subtypes such as glioblastoma, pancreatic, bladder, breast and rectal cancers	Clinical, mRNA, miRNA, CNV, methylation, methylation, miRNA, splicing, lncRNA, and mutation	https://www.cancer.gov/ccg/research/genome-sequencing/tcga	Cancer genome atlas (TCGA) generates genomics data, while GDC is the platform that hosts and shares not only TCGA data but also other cancer genomics datasets, promoting data accessibility and collaboration in the cancer research community.
UK Biobank (Bycroft et al., 2018)	Population-based cohort study with over 500,000 participants, collecting extensive health and genetic data	Clinical, genetic, imaging, lifestyle, and environmental data	https://www.ukbiobank.ac.uk/	UK Biobank is a large-scale prospective cohort study that aims to improve the prevention, diagnosis, and treatment of a wide range of illnesses, including cancer, by providing a rich resource for researchers to study the complex interplay between genetic, environmental, and lifestyle factors.

A deeper analysis of existing survival predictors reveals that among the 90 studies 58 utilized publicly accessible data from three key databases: the Cancer Genome Atlas Program (TCGA) (Tomczak et al., 2015), NCI Genomic Data Commons (GDC) (Jensen et al., 2017), and the Gene Expression Omnibus (GEO) (Clough and Barrett, 2016; Chai et al., 2021a; Hu Q. et al., 2021; Poirion et al., 2021; Zhao L. et al., 2021; Han et al., 2022; Jiang et al., 2022; Redekar et al., 2022; Wu and Fang, 2022; Wu et al., 2022; Zhang R. et al., 2022). Apart from public databases, there also exist private databases that have been utilized in existing survival prediction studies (Vahabi et al., 2021; Feng et al., 2022; Miao et al., 2022; Richard et al., 2022; Chauhan et al., 2023; Lee et al., 2023; Moreno-Sanchez, 2023). However, these private databases often restrict data access and may require extensive research proposals for data retrieval. Among these databases commonly used databases are Heidelberg University Hospital (Jung et al., 2023), COMBO-01 (Zhou et al., 2023), Life cohort (Unterhuber et al., 2021), and UNOS (Kantidakis et al., 2020; Raju and Sathyalakshmi, 2023). The reliance on private databases for survival prediction creates significant hurdles for research in several ways (Raufaste-Cazavieille et al., 2022). Firstly, limited accessibility to such data impedes the reproducibility and verification of study findings by other researchers, hindering the validation and robustness of predictive models (Misra et al., 2019). Secondly, the lack of transparency and standardized access procedures for private datasets introduces challenges in benchmarking and comparing different survival prediction models (Raufaste-Cazavieille et al., 2022). Lastly, the exclusivity of private databases may contribute to a potential bias in research outcomes, as the diversity and representativeness of the data are often compromised which impacts the generalizability of survival predictions to broader patient cohorts (Boffa et al., 2021).

Public access to databases enables researchers to create survival benchmark datasets that fosters the development of survival prediction models (Liu et al., 2018; Rahimi et al., 2023). However, many researchers develop datasets without making them public which hinders transparency and the broader scientific community progress (Weston et al., 2019). The lack of shared data and presence of multiple datasets associated with a single disease pose a notable challenge in survival prediction. For instance, it hinders the establishment of standardized testing and benchmarking procedures for newly proposed survival prediction methods, leading to ambiguities in identifying the most advanced techniques (Wissel et al., 2022). Moreover, recognizing the need for standardization in benchmarking survival prediction models, Wissel et al. (2022) introduced benchmark survival datasets tailored for both individual cancer subtypes and pan-cancer settings. These datasets are accessible at https://survboard.vercel.app/, contributing to a more uniform and transparent benchmarking framework within the survival prediction landscape. Particularly, here we emphasize the use of these datasets for benchmarking in addition to newly created datasets to have unified benchmarking for cancer-specific survival prediction models.

### 5.3 RQ V, VI: survival prediction data modalities and utilization of their combinations for disease and survival endpoints specific predictors development

Following the objective of research question V, the primary focus of this section is to investigate and provide a comprehensive summary of the various data modalities utilized in the development of diverse survival predictors. To address research question V, it describes the distribution of data modalities across predictors associated with four distinct survival endpoints, and 44 different diseases. Furthermore, in response to research question VI, it furnishes information regarding the specific clinical features utilized by various survival prediction studies.

Out of 90 different studies, data modalities details of only 84 studies are available. Within this subset, 27 studies exclusively used clinical data, 39 studies utilized multiomics data, and 16 studies investigated the combined potential of both clinical and multiomics data modalities. Moreover, based on characteristics of molecular information omics data is generally categorized into nine different classes namely gene expression (mRNA), micro RNA (miRNA), methylation, copy number variation (CNV), whole exome sequencing (WES), long noncoding RNA (lncRNA), mutation, metabolic, and proteomics. The specifics of different predictors, in terms of variations in the combinations of clinical and various omics data modalities, are outlined in Table 5. Among 55 survival prediction studies based on multiomics, 49 studies utilized different combinations of four distinct omics types: mRNA, methylation, miRNA, and CNV (Baek and Lee, 2020; Jiang et al., 2020; Li et al., 2020; Tan et al., 2020; Tong D. et al., 2020; Tong L. et al., 2020; Yang Q. et al., 2020; Chai et al., 2021a; Hira et al., 2021; Hu Q. et al., 2021; Owens et al., 2021; Tong et al., 2021; Zhang X. et al., 2021; Zhang Z.-S. et al., 2021; Zhao L. et al., 2021; Bhat and Hashmy, 2023; Ellen et al., 2023; Hao et al., 2023). Only seven studies utilized additional modalities such as whole exome sequencing (WES) (Baek and Lee, 2020; Jiang et al., 2022), long coding RNA (lncRNA) (Jiang et al., 2022), proteomics (Tan et al., 2020; Malik et al., 2021; Unterhuber et al., 2021; Richard et al., 2022; Pellegrini, 2023), and mutation data (Tan et al., 2020; Malik et al., 2021; Unterhuber et al., 2021; Pellegrini, 2023).

**Table 5:** Distribution of data modalities across diverse surival prediction studies.

**Data modality**	**References**																																	
	**Pellegrini (2023)**	**Jung et al. (2023)**	**Qian et al. (2023)**	**Chauhan et al. (2023)**	**Li et al. (2023)**	**Moreno-Sanchez (2023)**	**Wang J. et al. (2023)**	**Zhou et al. (2023)**	**Zhang et al. (2023)**	**Hao et al. (2023)**	**Lee et al. (2023)**	**Wang X. et al. (2023)**	**Zhang S. et al. (2022)**	**Manganaro et al. (2023)**	**Benkirane et al. (2023)**	**Othman et al. (2023)**	**Bhat and Hashmy (2023)**	**Fan et al. (2023)**	**Willems et al. (2023)**	**Ellen et al. (2023)**	**Yin et al. (2022)**	**Richard et al. (2022)**	**Wang et al. (2022)**	**Zhang J. Z. et al. (2022)**	**Miao et al. (2022)**	**Li et al. (2021)**	**Zhang Z.-S. et al. (2021)**	**Zhou et al. (2021)**	**Zhang X. et al. (2021)**	**Zheng et al. (2021)**	**Li et al. (2020)**	**Kazerooni et al. (2021)**	**Lv et al. (2020)**	**Jiang et al. (2020)**	**Zarean Shahraki et al. (2023)**	**Doja et al. (2020)**	**Pant et al. (2023)**	**Hu S. et al. (2021)**	**Eckardt et al. (2023)**	**Zhu et al. (2023)**	**Rahman et al. (2023)**
Clinical	✓	✓	✓	✓	✓	✓	✓	✓	×	×	✓	×	✓	✓	×	✓	✓	✓	×	✓	×	×	×	×	✓	✓	×	×	×	×	×	✓	×	×	✓	✓	✓	✓	✓	✓	✓
mRNA	✓	×	×	×	×	×	×	×	×	✓	✓	✓	✓	✓	✓	✓	✓	✓	✓	✓	✓	×	✓	✓	×	✓	✓	✓	✓	×	✓	×	✓	✓	×	×	×	×	×	×	×
miRNA	×	×	×	×	×	×	×	×	✓	✓	×	×	×	×	×	×	✓	✓	✓	✓	×	×	×	×	×	✓	✓	×	✓	✓	×	×	✓	✓	×	×	×	×	×	×	×
Methylation	✓	×	×	×	×	×	×	×	×	✓	×	×	×	×	✓	×	✓	×	×	✓	×	×	✓	✓	×	✓	✓	×	✓	×	✓	×	✓	✓	×	×	×	×	×	×	×
CNV	×	×	×	×	×	×	×	×	×	×	✓	×	✓	✓	✓	✓	✓	✓	×	×	×	×	×	×	×	×	×	✓	×	✓	×	×	×	✓	×	×	×	×	×	×	×
Proteomics	✓	×	×	×	×	×	×	×	×	×	×	×	×	×	×	×	×	×	×	×	×	✓	×	×	×	×	×	×	×	×	×	×	×	×	×	×	×	×	×	×	×
Mutation	×	×	×	×	×	×	×	×	×	×	×	×	✓	✓	×	×	×	×	×	×	×	×	×	×	×	×	×	×	×	✓	×	×	×	×	×	×	×	×	×	×	×
Metabolic	×	×	×	×	×	×	×	×	×	×	×	×	×	×	×	×	×	×	×	×	×	✓	×	×	×	×	×	×	×	×	×	×	×	×	×	×	×	×	×	×	×
lncRNA	×	×	×	×	×	×	×	×	×	×	×	×	×	×	×	×	×	×	×	×	×	×	×	×	×	×	×	×	×	×	×	×	×	×	×	×	×	×	×	×	×
WES	×	×	×	×	×	×	×	×	×	×	×	×	×	×	×	×	×	×	×	×	×	×	×	×	×	×	×	×	×	×	×	×	×	×	×	×	×	×	×	×	×
**Data modality**	**References**																																	
	Lin et al. (2022)	Feng et al. (2022)	Bichindaritz and Liu (2022)	Wu et al. (2022)	Jiang et al. (2022)	Zhang R. et al. (2022)	Wu and Fang (2022)	Hathaway Q. A. et al. (2021)	Pawar et al. (2022)	Redekar et al. (2022)	Han et al. (2022)	Zeng et al. (2021)	Unterhuber et al. (2021)	Hu Q. et al. (2021)	Poirion et al. (2021)	Kantidakis et al. (2020)	Zhao L. et al. (2021)	Chai et al. (2021a)	Tang et al. (2021)	Vahabi et al. (2021)	Malik et al. (2021)	Owens et al. (2021)	Hira et al. (2021)	Tong et al. (2021)	Du et al. (2020)	Yu et al. (2020)	Yang Q. et al. (2020)	Wang et al. (2020)	Yang H. et al. (2020)	Baek and Lee (2020)	Tong L. et al. (2020)	Tong D. et al. (2020)	Tan et al. (2020)	Lee et al. (2020)	Palmal et al. (2023)	Lun et al. (2024)	Wang Q. et al. (2023)	Lang et al. (2023)	Farahani et al. (2023)	Fala and Osman (2023)	Tarango et al. (2023)
Clinical	✓	✓	✓	×	×	×	×	✓	×	✓	×	✓	✓	×	×	✓	✓	×	×	×	×	×	×	×	×	×	×	×	×	✓	×	✓	×	×	✓	×	×	×	×	×	×
mRNA	×	×	✓	✓	✓	✓	✓	×	✓	✓	✓	×	×	✓	✓	×	✓	✓	✓	✓	✓	✓	✓	✓	✓	✓	✓	✓	✓	✓	✓	✓	✓	✓	✓	×	×	×	×	×	×
miRNA	×	×	×	×	✓	×	×	×	×	×	×	×	×	✓	✓	×	✓	✓	×	×	×	✓	✓	✓	×	×	×	✓	✓	✓	✓	✓	✓	✓	×	×	×	×	×	×	×
Methylation	×	×	✓	✓	✓	×	×	×	×	✓	✓	×	×	✓	✓	×	✓	✓	×	✓	✓	✓	✓	✓	×	✓	×	✓	✓	✓	✓	✓	✓	✓	×	×	×	×	×	×	×
CNV	×	×	×	×	×	✓	✓	×	×	✓	✓	×	×	✓	×	×	✓	✓	×	×	✓	×	✓	✓	✓	×	×	×	×	×	✓	×	×	✓	✓	×	×	×	×	×	×
Proteomics	×	×	×	×	×	×	×	×	×	×	×	×	✓	×	×	×	×	×	×	×	✓	×	×	×	×	×	×	×	×	×	×	×	✓	×	×	×	×	×	×	×	×
Mutation	×	×	×	×	×	×	×	×	×	×	✓	×	×	✓	×	×	×	×	×	×	✓	×	×	×	×	×	✓	×	×	×	×	×	×	×	×	×	×	×	×	×	×
Metabolic	×	×	×	×	×	×	×	×	×	×	×	×	×	×	×	×	×	×	×	×	×	×	×	×	×	×	×	×	×	×	×	×	×	×	×	×	×	×	×	×	×
lncRNA	×	×	×	×	✓	×	×	×	×	×	×	×	×	×	×	×	×	×	×	×	×	×	×	×	×	×	×	×	×	×	×	×	×	×	×	×	×	×	×	×	×
WES	×	×	×	×	✓	×	×	×	×	×	×	×	×	×	×	×	×	×	×	×	×	×	×	×	×	×	×	×	×	✓	×	×	×	×	×	×	×	×	×	×	×

**Figure 6:**
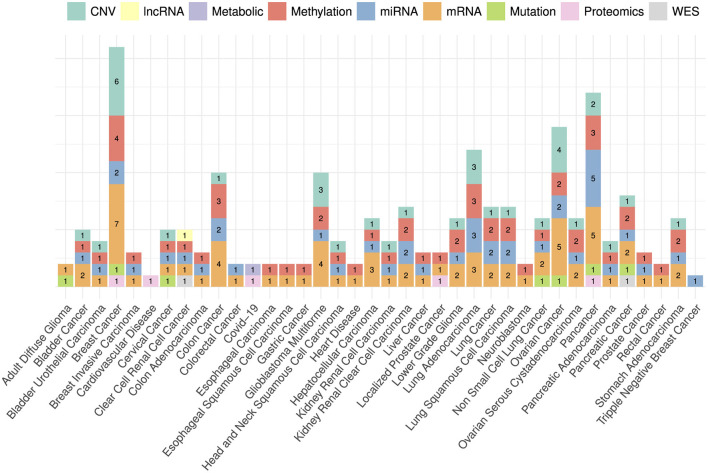
Distribution of omics data modalities across a diverse set of diseases. Bar heights represent the counts of each data modality with respect to disease specific published research papers. For instance, CNV has been used in six papers related to breast cancer, mRNA has been used in seven breast cancer papers and so on.

The choice of omics type hinges on the specific disease under investigation, as indicated by the disease-wise distribution of omics types in Figure 6. Out of nine omics types, mRNA, CNV, miRNA, and methylation have been the most commonly utilized modalities for 33 cancer subtypes i.e., breast cancer (Tong L. et al., 2020; Malik et al., 2021; Zhou et al., 2021; Wu and Fang, 2022; Zhang J. Z. et al., 2022; Hao et al., 2023; Othman et al., 2023; Zhang et al., 2023), pan-cancer (Tan et al., 2020; Poirion et al., 2021; Zhang X. et al., 2021; Zheng et al., 2021; Redekar et al., 2022; Yin et al., 2022; Fan et al., 2023), colon cancer (Lv et al., 2020; Tong D. et al., 2020; Yang H. et al., 2020; Zhang J. Z. et al., 2022; Lee et al., 2023), lung adenocarcinoma (Jiang et al., 2020; Lee et al., 2020; Bhat and Hashmy, 2023), and ovarian cancer (Hira et al., 2021; Tong et al., 2021; Zhao L. et al., 2021; Pawar et al., 2022; Wu and Fang, 2022; Zhang S. et al., 2022). In addition, mutation data has been utilized for seven cancer subtypes namely, adult diffuse glioma (Yang Q. et al., 2020), breast cancer (Malik et al., 2021), cervical cancer (Hu Q. et al., 2021), non-small cell lung cancer (Manganaro et al., 2023), ovarian cancer (Zhang S. et al., 2022), and pancreatic cancer (Han et al., 2022). Among 10 data modalities, three modalities namely, proteomic, lncRNA and WES have been utilized the least having limited applicability to clear renal cell cancer (Jiang et al., 2022), pancreatic cancer (Baek and Lee, 2020), breast cancer (Malik et al., 2021), localized prostate cancer (Pellegrini, 2023), and pan-cancer (Zheng et al., 2021). In terms of other diseases i.e., COVID-19 and heart diseases, proteomics, methylation, mRNA, metabolic, and methylation have been the only omics types utilized for survival prediction (Unterhuber et al., 2021; Vahabi et al., 2021; Richard et al., 2022).

**Figure 7:**
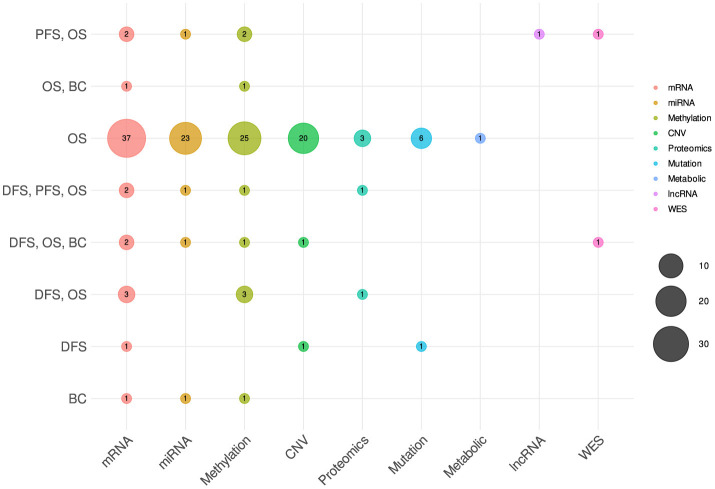
Distribution of different omics modalities with respect to survival endpoints.

The variability in omics-type selection is not solely bound to diseases but notably varies across a wide spectrum of survival endpoints. Figure 7 shows the counts of different omics types that have been utilized for different survival endpoints prediction. In the context of OS prediction, mRNA, miRNA, methylation, and CNV have been primarily utilized in more than 31 studies, with 10 studies based on proteomics, mutation, and metabolic data. However, in terms of DFS and PFS the selection of omics types appears less distinct. These endpoints have been frequently studied in conjunction with OS, predominantly utilizing mRNA, miRNA, and methylation data. This combination suggests a commonality in the predictive factors across these survival endpoints, indicating potential interconnections or shared biological processes.

Clinical data modality has been utilized in 42 different studies. However, in this modality number of features varied from study to study and it is still unclear which particular set of features is most important. To perform an in-depth analysis, which study utilized which subset of features across diverse cancer subtypes and heart diseases, a comprehensive collection of clinical features is presented in Table 6. In order to better understand and discern the trends in clinical features across diverse diseases, hereby they are placed in seven different categories i.e., demographic features (6), disease-specific clinical markers (71), treatment-related features (17), laboratory and biomarkers (48), comorbidity and lifestyle factors (18), and other factors (15).

**Table 6:** Diverse collection of clinical features utilized in various survival prediction studies.

**References**	**Disease**	**Total**	**Features (generic)**
Qian et al. (2023)	Atherosclerotic cardiovascular disease (ASCVD)	39	Height, weight, waist and hip circumference, blood pressure, B-ultrasound, heart rate, hypertension, prehypertension, pulse pressure difference, body mass index, body obesity index, waist to hip ratio, composite index triglyceride, blood glucose index, fat accumulation product index, lipoprotein binding index, atherosclerosis index, atherogenic index of plasma, low high density lipoprotein ratio, bilirubin composite index, family history of diseases of diabetes or ASCVD, smoking, alcohol consumption, fasting blood glucose, triglyceride, high-density lipoprotein cholesterol, total cholesterol, low-density lipoprotein cholesterol, diabetes, fatty liver, blood glucose index, fat accumulation product index, lipoprotein binding index, atherosclerosis index, AIP, low–high-density lipoprotein ratio and bilirubin composite index
Pellegrini (2023)	Localized prostate cancer	24	Prostate-specific antigens, Gleason primary score, tumor stage expression levels for NF2 and CDKN1B
Jung et al. (2023)	Gastroesophageal cancer	117	Biometric variables, past medical history of diseases, tumor diagnosis, cTNM classification, histology, neoadjuvant therapy, time between diagnosis and resection, type of operation, extent of resection, anatomical reconstruction, duration of surgery, intraoperative complication, blood loss and transfusion, days on ICU and ward, postoperative complications, pTNM classification, lymph node ratio, grading, r status, histology, post-discharge problems
Chauhan et al. (2023)	Bladder cancer	17	Gender, median age, ethnicity, smoking history, initial tumor stage, neoadjuvant chemotherapy received, histology, pathology, pathologic complete response, smoking history, pack years, body mass index, hemotocrit, urine cfDNA, variant allele frequency, inferred tumor mutational burden, tumor fraction
Li et al. (2023)	Lymphoma	18	Sex, age at diagnosis, ethnic, medical insurance, Ann Arbor stage, pathological type, b symptoms, surgery, radiotherapy, chemotherapy, targeted therapy, immunotherapy, LDH, β2-microglobulin, platelet, lymphocyte, albumin globulin ratio and C reactive protein
Lee et al. (2023)	Colon cancer	7	Age, sex, AJCC stage, prognostic information such as alive, deceased, disease free and recurrence
Wang X. et al. (2023)	Hepatocellular carcinoma	24	Age, gender, ALT, main tumor size, multinodular, cirrhosis, TNM stage, BCLC stage, CLIP stage, tumor grade, TMB, stromal score, immune score, ESTIMATE score, risk score, CNLC stage, hepatitis B, Lymph node invasion, vascular invasion, perineural invasion, albumin, AFP, CEA and CA199
Manganaro et al. (2023)	Non-small cell lung cancer	6	Histology, gender, age, pathological staging, DFS, and smoking status related features
Othman et al. (2023)	Breast cancer	25	Age at diagnosis, tumor size, tumor stage, lymph nodes, examined positive neoplasm, histologic grade, histological type, ER status, PR status, HER2 SNP6 state, type of treatment, the patient received survival status and time, inferred menopausal state, overall survival, HER2 SNP6 state, treatment and patients vital status
Moreno-Sanchez (2023)	Heart failure	13	Age, anemia, high blood pressure, creatinine phosphokinase, diabetes, ejection fraction, sex, platelets, serum creatinine, serum sodium, smoking, time follow up period and death event
Wang J. et al. (2023)	Metastatic breast cancer	10	Age at diagnosis, mean age, molecular classification (luminal, triple-negative), de novo metastasis, number of metastatic sites, visceral metastases, adjuvant chemotherapy, adjuvant radiotherapy, adjuvant endocrine therapy and previous endocrine therapy
Ellen et al. (2023)	Non-small lung cancer	11	Age, sex, tumor, volume, primary diagnosis, prior malignacy, synchronous malignancy, pathological stage, staging tumor, staging lymph nodes, staging metastasis, no. of pack-years smoked
Miao et al. (2022)	Nasopharyngeal carcinoma	17	Age, stage, sex, ethnicity, marriage, occupation, pathological, transfer information, radiotherapy, chemical therapy, targeted therapy, EBV, BQ, LAR, NLR and PLR
Feng et al. (2022)	Cardiovascular	8	Age, age groups, sex, region of residence, number of Charlson comorbidities, Charlson comorbidities, lab test results(LDL-cholesterol, blood glucose, eGFR, HbA1c, at least one lab test), features related to medications
Bichindaritz and Liu (2022)	Esophageal carcinoma	3	Vital status, days to death and days to last follow up
Redekar et al. (2022)	Glioblastoma multiforme	9	Age, gender, diagnosis method, treatment history, Karnofsky score, performance score, radiation therapy, duration of survival and death status
Unterhuber et al. (2021)	Cardiovascular disease	12	Age, sex, body mass index, smoking status, systolic and diastolic blood pressure, current smoker, total cholesterol, HDL cholesterol, triglycerides, lipid lowering drug dose, antihypertensive drug use and median follow-up time
Hathaway Q. A. et al. (2021)	Atherosclerosis	33	Age, albumin/creatinine ratio, BMI, cholesterol, diabetes, educational status, family history of heart diseases, gender, HDL, hyperlipidemia, hypertension, income category, LDL, mean diastolic and systolic blood pressure, metabolic syndrome, smoking in past years, statin use, triglycerides, minutes walking per week, c reactive protein, D dimer, factor VIII, fibrinogen antigen, homocysteine, interleukin-6, plasmin antiplasmin, pericardial fat deposition, coronary artery calcium score, left ventricular area and left ventricular ejection fraction
Zhao L. et al. (2021)	Multiple cancer subtypes	3	Age, Overall Survival time, Status
Li et al. (2021)	Prostate cancer	4	Age at diagnosis, clinical tumor stage (T1(a-c),T2(a-c), T3(a-b),T4), NA Gleason score and preoperative PSA
Kantidakis et al. (2020)	Liver transplantation	97	52 donor and 45 liver recipient characteristics, unique encrypted person Id, unique encrypted donor Id, candidate listing center, OPO serving, transplant center, transplant date, graft failure date, cohort censoring date, death date, graft follow up date, age, gender, race, ethnicity, socioeconomic status and education level, smoking history, alcohol consumption, physical activity level, cocaine or other drug history, blood type, etiology (cause of disease), laboratory measurements for arginine, serum creatinine, serum sodium, total bilirubin
Baek and Lee (2020)	Pancreatic adenocarcinoma (PAAD)	7	Sex, grade, AJCC cancer stage, smoking history, treatment outcome, age, primary site
Tong D. et al. (2020)	Colon cancer	7	Gender, survival status, survival time, TNM stage, age at initial diagnosis

A closer look at the clinical features across diverse diseases reveals a consistent set of fundamental demographic features i.e., age and gender which are prevalent in nearly all studies (Hathaway Q. A. et al., 2021; Unterhuber et al., 2021; Feng et al., 2022; Redekar et al., 2022; Li et al., 2023; Wang X. et al., 2023). Beyond demographic features, disease-specific features also play critical role for disease-specific survival prediction. For instance, cancer-related studies invariably focus on tumor stage, histological type, and treatment specifics, underlining the critical role of disease-specific clinical markers in prognosis (Lee et al., 2023; Pellegrini, 2023).

Treatment-related features such as chemotherapy, radiotherapy, and immunotherapy, are particularly evident in cancer subtypes specific studies which reflect the profound influence of therapeutic interventions on survival outcomes (Othman et al., 2023; Wang X. et al., 2023). Moreover, the recurrent inclusion of lifestyle and comorbidity factors ranging from smoking history and BMI to hypertension and diabetes across multiple diseases underlines their pervasive impact on prognostic modeling (Hathaway Q. A. et al., 2021; Bhat and Hashmy, 2023). These lifestyle and comorbidity features show the complex relationship between individual health choices and their potential influence on survival outcomes.

### 5.4 RQ VII: feature engineering trends across data modalities and disease-specific survival predictors

This section addresses research question VII by investigating the application of feature engineering methods in survival prediction studies across a variety of diseases. This will help researchers to analyze and understand trends of feature engineering techniques in disease or endpoint specific survival prediction pipelines. Additionally, it delves into the trends in diverse feature engineering methods and their relevance to clinical and multiomics data modalities. This investigation aims to reveal trends and patterns in the dynamic interplay between feature engineering methods and the specific characteristics of different data modalities, and survival endpoints.

Table 7 illustrates 30 different feature engineering methods that have been utilized in diverse survival prediction studies. These methods are broadly categorized into five categories, namely supervised methods, incorporating L1 regularized Cox regression (Qian et al., 2023), RSF algorithm (Qian et al., 2023), Cox regression (Zhang S. et al., 2022), least absolute shrinkage and selection operator (lasso) regression (Abdelhamid et al., 2022), cascaded Wx (Yin et al., 2022), recursive feature elimination (Wang et al., 2022), Boruta (Jiang et al., 2022), Akaike information criterion (AIC) regression (Zeng et al., 2021), variance (Zhao L. et al., 2021), lasso analysis (Tang et al., 2021), multivariate regression (Tang et al., 2021), Chi-squared (Moreno-Sanchez, 2023), mutual information (Moreno-Sanchez, 2023), and ANOVA (Lv et al., 2020; Moreno-Sanchez, 2023). Additionally, Network based methods include network based stratification (NBS) (Shetty et al., 2023), weighted correlation network analysis (WGCNA) (Wang X. et al., 2023), canonical correlation analyses (CCA) (Wang J. et al., 2023), patient similarity networks (Wang et al., 2022), and neighborhood component analysis (NCA) (Malik et al., 2021). Dimensionality reduction methods include non-negative matrix factorization (NMF) (Tang et al., 2021), autoencoders (AEs) (Benkirane et al., 2023), variational autoencoders (VAEs) (Owens et al., 2021), principal component analysis (PCA) (Lv et al., 2020), and dominant effect of the cancer driver genes (DEOD) (Amgalan and Lee, 2015; Lee et al., 2023). Moreover, clustering methods comprise Kruskal-Wallis and Gaussian clustering (Poirion et al., 2021), hierarchical clustering (Chai et al., 2021a), and Guassian clustering (Poirion et al., 2021). In addition, to deal with clinical data, Palmal et al. (2023) showed the application of Tab-transformer for feature extraction.

**Table 7:** Diverse feature engineering methods for survival prediction.

**Method name**	**References**
L1 regularized Cox regression	Qian et al., 2023
RSF algorithm	Qian et al., 2023
Cox regression	Zhang S. et al., 2022; Qian et al., 2023
Network-Based Stratification (NBS) for data integration	Shetty et al., 2023
Weighted correlation network analysis (WGCNA)	Wang X. et al., 2023
Canonical correlation analyses (CCA)	Wang J. et al., 2023
Least Absolute Shrinkage and Selection Operator (lasso) regression modeling	Abdelhamid et al., 2022; Eckardt et al., 2023
Cascaded Wx	Yin et al., 2022
Recursive feature elimination	Wang et al., 2022; Eckardt et al., 2023
Patient similarity networks	Wang et al., 2022
Boruta	Jiang et al., 2022
Akaike Information Criterion (AIC) regression	Zeng et al., 2021
Kruskal-Wallis and Gaussian clustering	Poirion et al., 2021
Variance	Zhao L. et al., 2021
Non-negative matrix factorization (NMF)	Tang et al., 2021
Lasso analysis	Tang et al., 2021
Multivariate regression	Tang et al., 2021
PCA	Lv et al., 2020; Chai et al., 2021a; Jiang et al., 2022; Zhang S. et al., 2022
ANOVA	Lv et al., 2020; Moreno-Sanchez, 2023
Chi-squared	Eckardt et al., 2023; Moreno-Sanchez, 2023
Mutual information	Moreno-Sanchez, 2023
Hierarchical clustering	Tong D. et al., 2020; Chai et al., 2021a
Neighborhood component analysis (NCA)	Malik et al., 2021
DEOD	Lee et al., 2023
Minimal-redundancy-maximal-relevance criterion (MRMR)	Palmal et al., 2023
Graph convolution Networks	Palmal et al., 2023
Tab-transformer	Pant et al., 2023
Transformer	Hu S. et al., 2021
Variational autoencoders	Tong L. et al., 2020; Hira et al., 2021; Zhang X. et al., 2021; Benkirane et al., 2023; Bhat and Hashmy, 2023
Autoencoders	Baek and Lee, 2020; Jiang et al., 2020, 2022; Li et al., 2020; Lv et al., 2020; Wang et al., 2020; Yang H. et al., 2020; Owens et al., 2021; Wu and Fang, 2022

A comprehensive analysis of feature engineering methods across a range of disease-specific survival prediction studies unveils that supervised methods, such as Cox regression, L1 regularized Cox regression, and RSF algorithm, have been prevalent in diseases like ASCVD, trauma, and ovarian cancer (Abdelhamid et al., 2022; Zhang S. et al., 2022). On the other hand, network based methods including NBS and WGCNA, have been applied in diseases like KIRP, and hepatocellular carcinoma, which shows the significance of network structures in certain medical contexts (Wang X. et al., 2023). Univariate analyses, including ANOVA, chi-squared, and univariate Cox regression, have been prevalent in diseases such as pancreatic cancer and heart failure, underscoring the significance of statistical testing in identifying relevant features (Moreno-Sanchez, 2023; Zhou et al., 2023). Furthermore, dimensionality reduction methods such as PCA, and NMF have been consistently used across various diseases namely, ovarian cancer (Zhang S. et al., 2022), lower grade glioma (Wu et al., 2022), colon adenocarcinoma (Lv et al., 2020), bladder and breast cancers (Tang et al., 2021; Lin et al., 2022). In addition, the potential of AEs, and VAEs have also been explored in diseases like glioblastoma multiforme, breast cancer, pan-cancer, and Lung Adenocarcinoma for feature integration and dimensionality reduction (Benkirane et al., 2023; Bhat and Hashmy, 2023; Hao et al., 2023).

While feature engineering methods exhibit specificity tailored to distinct diseases, their efficacy is influenced by the inherent characteristics of the utilized data (Jiang et al., 2017). This raises the pertinent question of which particular feature engineering method proves most effective in the context of clinical and multiomics datasets. A thorough analysis of feature engineering methods and their applicability with respect to clinical and multiomics datasets reveals that methods like Cox regression, CCA, AIC, and ANOVA have been quite widely utilized in studies involving only clinical data (Zeng et al., 2021; Moreno-Sanchez, 2023; Qian et al., 2023; Wang J. et al., 2023). These methods have been applied to clinical data for multiple reasons for instance, such methods are interpretable which is important to gain meaningful insights for healthcare professionals (Jiang, 2022). Clinical data is always multifactorial, which means that multiple features of the data can lead to a specific event, and methods like ANOVA are quite efficient in analyzing such contributors (Azizi et al., 2022). Although, such models have shown promising performance with clinical data, yet one of the drawbacks of such models is their inability to handle non-linear data which is the case in terms of multiomics data (Cleves, 2008). Considering similar limitations, multiple methods such as cascaded wx (Yin et al., 2022), RFI (Wang et al., 2022), PSN (Jiang et al., 2022), NMF (Tang et al., 2021), Boruta (Jiang et al., 2022), PCA (Chai et al., 2021a) variance (Zhao L. et al., 2021), DEOD (Lee et al., 2023), have been utilized to handle multiomics to capture important interactions among the features and to integrate cross modalities properly. Particularly, here methods such as AEs and VAEs play a significant role and recent studies also show a growing interest in using such methods for dimensionality reduction and feature integration by such methods for multiomics and clinical datasets i.e., AEs (Baek and Lee, 2020; Jiang et al., 2020, 2022; Li et al., 2020; Lv et al., 2020; Wang et al., 2020; Yang H. et al., 2020; Owens et al., 2021; Wu and Fang, 2022), and VAEs (Tong L. et al., 2020; Hira et al., 2021; Zhang X. et al., 2021; Benkirane et al., 2023).

Although the selection of a feature engineering method is tied to the characteristics of the disease and the nature of the data (Dong and Liu, 2018), there is no significant evidence to suggest that it is substantially impacted by survival endpoints such as DFS, PFS, BC, and OS. This assumption arises due to the absence of a consistent pattern in feature engineering method selection across different survival endpoints. Studies, such as Lv et al. (2020), Tang et al. (2021), and Manganaro et al. (2023), demonstrate a varied use of feature engineering techniques irrespective of the specific survival endpoints (DFS, PFS, BC, or OS). This lack of uniformity implies that feature engineering method selection is driven more by the unique characteristics of the data and disease than by the nature of the survival endpoint itself.

On the basis of various trends and patterns it can be concluded that for heart diseases, univariate analyses and supervised feature engineering methods have been utilized. Conversely, in terms of cancer subtypes a mixture of dimensionality reduction methods is observed with a recent trend toward the AEs. In terms of survival datasets, the prime focus has been to use supervised methods for clinical data and multiple dimensionality reduction methods for multiomics data. Moreover, there are no conclusive remarks that feature engineering methods get affected by the survival endpoints, as the current literature also suggests a varied use of feature engineering methods regardless of the survival endpoints.

### 5.5 RQ VIII: survival prediction methods insights and distribution across disease types and survival endpoints

In pursuit of addressing research question VIII, this section presents an overview and insights about statistical, ML, and DL algorithms that have been utilized in existing survival prediction pipelines. It succinctly examines their emerging trends across diseases and survival endpoints. This exploration aims to empower researchers in identifying gaps within disease-specific and survival endpoint-focused studies, ultimately contributing to the enhancement of survival predictive pipelines.

Table 8 provides information about 44 diseases and the corresponding survival prediction algorithms utilized in these diseases. A deeper analysis of Table 8 shows that Cox-PH and lasso Cox-PH models have been extensively utilized for disease specific survival prediction i.e., ASCVD (Hathaway Q. A. et al., 2021; Qian et al., 2023), bladder cancer (Chai et al., 2021a; Tang et al., 2021), colorectal cancer (Tong D. et al., 2020; Yang H. et al., 2020; Zhang J. Z. et al., 2022; Lee et al., 2023), hepatocellular carcinoma (Owens et al., 2021; Zhang R. et al., 2022; Wang X. et al., 2023), ovarian cancer (Hira et al., 2021; Pawar et al., 2022; Wu and Fang, 2022; Zhang S. et al., 2022), lung adenocarcinoma (Bhat and Hashmy, 2023), heart failure (Moreno-Sanchez, 2023), HER2-negative metastatic breast cancer (Wang J. et al., 2023), pancreatic cancer (Baek and Lee, 2020; Zhou et al., 2023), trauma (Abdelhamid et al., 2022), nasopharyngeal carcinoma (Miao et al., 2022), triple-negative breast cancer (Zhang et al., 2023), lymphoma (Li et al., 2023), breast cancer (Chai et al., 2021a; Tang et al., 2021; Chauhan et al., 2023), ovarian cancer (Hira et al., 2021; Pawar et al., 2022; Wu and Fang, 2022; Zhang S. et al., 2022), and lower-grade glioma (Wu et al., 2022), cardiovascular disease (Unterhuber et al., 2021; Vahabi et al., 2021; Xu et al., 2021; Zeng et al., 2021; Feng et al., 2022), invasive ductal carcinoma (Lin et al., 2022), liver transplantation (Kantidakis et al., 2020), gastric cancer (Li et al., 2020), lung cancer (Jiang et al., 2020), esophageal squamous cell carcinoma (Yu et al., 2020), glioma (Yang Q. et al., 2020), and liver cancer (Wang et al., 2020). RSF has been employed in 13 studies for six diseases namely, ASCVD (Qian et al., 2023), bladder cancer (Chai et al., 2021a), gastrointestinal cancer (Jung et al., 2023), cervical cancer (Hu Q. et al., 2021), liver transplantation (Kantidakis et al., 2020), and heart failure (Moreno-Sanchez, 2023). DL model DeepSurv, has been utilized in five studies related to gastrointestinal cancer (Jung et al., 2023), ASCVD (Hathaway Q. A. et al., 2021), NSCLC (Zhang Z.-S. et al., 2021). On the other hand, in the analyzed survival predictive pipelines less frequently utilized methods are i.e., survival SVM (Yu et al., 2020; Abdelhamid et al., 2022; Manganaro et al., 2023), partial logistic regression (Lin et al., 2022; Lee et al., 2023), log hazard net (Lee et al., 2023; Majji et al., 2023), boosting (Wang et al., 2020; Feng et al., 2022), stepCox (Wang X. et al., 2023), elastic net (Manganaro et al., 2023), CNN-cox (Majji et al., 2023), DeepOmix (Majji et al., 2023), ordinal Cox-PH (Bichindaritz and Liu, 2022), DeepHit (Feng et al., 2022), and linear multitask logistic regression (MTLR) (Feng et al., 2022).

**Table 8:** Distribution of survival predictors across diverse diseases.

**References**	**Disease**	**Predictor**
Hathaway Q. A. et al. (2021), Qian et al. (2023)	ASCVD	Cox-PH, RSF, MTLR, Deepsurv neural network, lasso Cox-PH
Hao et al. (2023), Shetty et al. (2023)	KIRP	GeneNet, ANNs
Li et al. (2021), Pellegrini (2023)	Prostate cancer	Coherent Voting Network (CVN), Best Linear Unbiased Prediction (BLUP)
Jung et al. (2023)	Gastrointestinal cancer	DeepSurv, MTLR, Gompertz model, RSF
Zhang et al. (2023)	Tripple negative breast cancer	lasso Cox-PH
Chai et al. (2021a), Tang et al. (2021), Chauhan et al. (2023)	Bladder cancer	Cox-PH, RSF, CoxNet, and transfer learning-based CoxNet
Li et al. (2023)	Lymphoma	lasso-Cox-PH
Tong D. et al. (2020), Yang H. et al. (2020), Zhang J. Z. et al. (2022), Lee et al. (2023)	Colon cancer	Loghazard Net, partial logistic regression, Cox-PH
Owens et al. (2021), Zhang R. et al. (2022), Wang X. et al. (2023)	Hepatocellular carcinoma	Stepwise Cox (StepCox), SurvivalSVM, Cox-PH, CoxNet
Hira et al. (2021), Pawar et al. (2022), Wu and Fang (2022) Zhang S. et al. (2022)	Ovarian cancer	Cox-PH, Cox-Time, and DeepSurv with consensus training
Zhang Z.-S. et al. (2021), Ellen et al. (2023), Manganaro et al. (2023)	NSCLC	SVM, Elastic net and Cox-PH, CNN and ANN
Benkirane et al. (2023)	Multiple cancers	ANN
Tong L. et al. (2020), Malik et al. (2021), Wu and Fang (2022), Othman et al. (2023)	Breast cancer	CoxNet, Cox-PH, Cox-Time, and DeepSurv with consensus training, Loghazard Net, partial logistic regression
Bhat and Hashmy (2023)	Lung adenocarcinoma	Cox-PH, and lasso Cox-PH
Tan et al. (2020), Poirion et al. (2021), Zhang X. et al. (2021), Zheng et al. (2021), Zhao L. et al. (2021), Yin et al. (2022), Fan et al. (2023), Majji et al. (2023)	Pan-cancer	Survival neural network, CNN-Cox, Cox-PH, DeepOmix, lasso and group penalized Cox-PH, VAE based NN
Willems et al. (2023)	Colorectal cancer	Lasso Cox-PH
Moreno-Sanchez (2023)	Heart failure	Cox-PH, RSF
Wang J. et al. (2023)	HER2-negative metastatic breast cancer	Cox-PH
Baek and Lee (2020), Zhou et al. (2023)	Pancreatic cancer	Cox-PH, l2 regularized regression
Abdelhamid et al. (2022)	Trauma	RF, SVM for outcome prediction
Miao et al. (2022)	Nasophrngeal carcinoma	Cox-PH
Unterhuber et al. (2021), Vahabi et al. (2021), Xu et al. (2021), Zeng et al. (2021), Feng et al. (2022)	Cardiovascular disease	Survival outcome prediction based on naive Bayes, ANNs, and SVM, Logistic regression and XGboost. Survival prediction: Cox-PH, survival XGboost, DeepHit, DeepSurv, Cox-PH, Linear MTLR, and RSF
Richard et al. (2022)	COVID-19	SVM
Wang et al. (2022)	Neuroblastoma	Deep neural network (DNN)
Lin et al. (2022)	Invasive ductal carcinoma	Multivariate Cox two way stepwise regression
Bichindaritz and Liu (2022)	Stomach, Esophageal carcinoma and Ovarian serous cystadenocarcinoma	Bidirectional LSTM, ordinal Cox model network and auxiliary loss
Wu et al. (2022)	Lower grade glioma	Lasso Cox-PH
Jiang et al. (2022)	Renal cell carcinoma	Cox-PH
Du et al. (2020), Lee et al. (2020), Kazerooni et al. (2021), Redekar et al. (2022), Wu and Fang (2022)	Glioblastoma	Cox-PH, CoxNet, SVM and Cox-PH, lasso Cox-PH
Hu Q. et al. (2021)	Cervical cancer	RSF, and Cox-PH
Tong et al. (2021)	Ovarian and breast cancer	
Kantidakis et al. (2020)	Liver transplantation	RSF, Cox-PH, and partial logistic artificial neural networks (PLANN)
Lv et al. (2020)	Lung adenocarcinoma	
Li et al. (2020)	Gastric cancer	Lasso, univariate and multi-variate Cox-PH
Jiang et al. (2020)	Lung cancer	Cox-nnet
Yu et al. (2020)	Esophageal squamous cell carcinoma	Support vector machine, K-means clustering
Yang Q. et al. (2020)	Glioma	Cox regression
Wang et al. (2020)	Liver cancer	XGBoost for subtype classification, and Cox-PH for survival prediction

**Figure 8:**
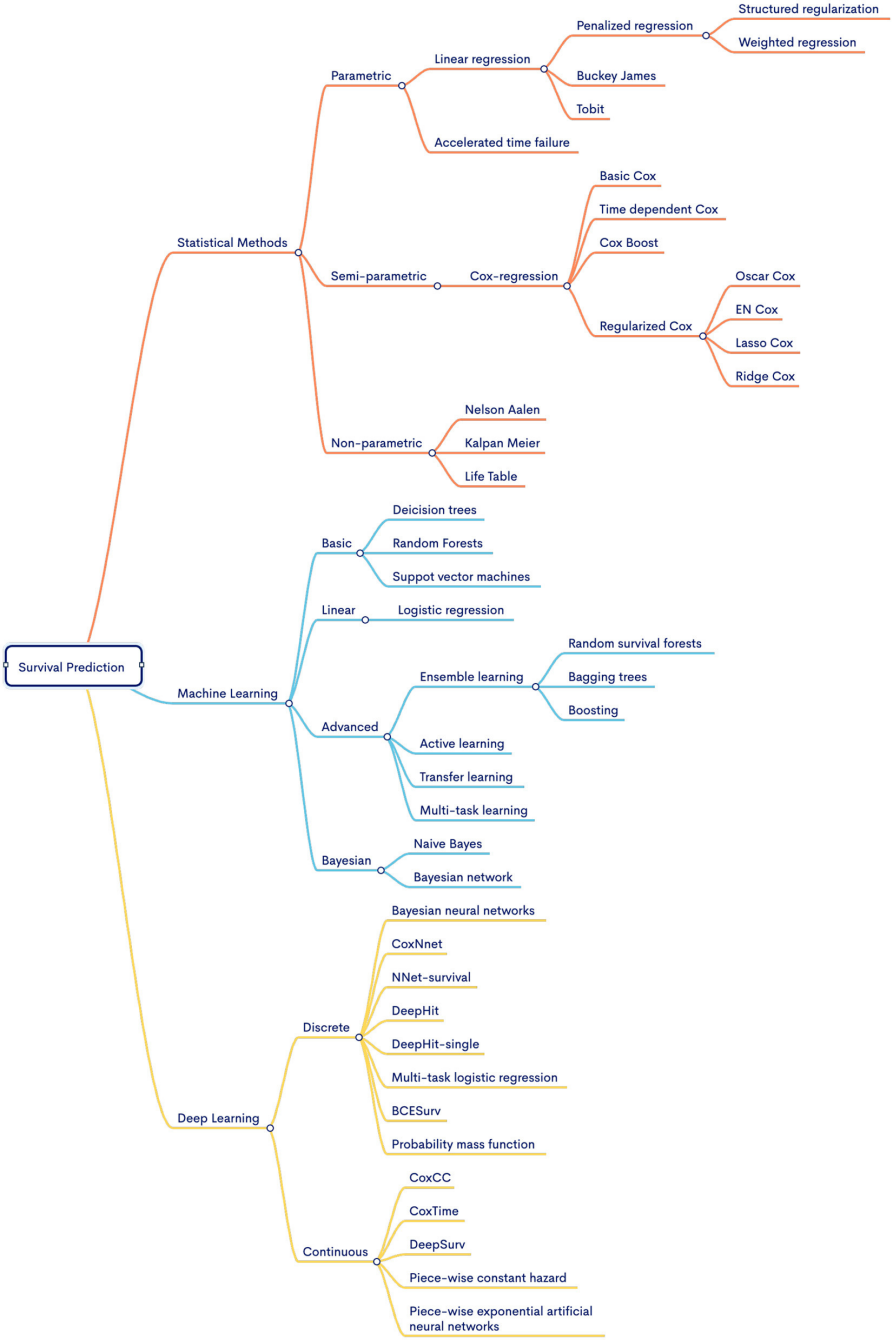
Hierarchal illustration of survival prediction methods under three different categories.

Furthermore, Supplementary Table S3 provides details about predictors distribution with respect to survival endpoints. A detailed analysis reveals, out of 90 predictors, 47, 8, 1, and 6 models have been utilized for OS, DFS, PFS, and BC survival endpoints, respectively. Unlike disease-specific predictors, here a mixture of methods is utilized and no particular trend exists. To provide high-level overview of multiple methods that have been utilized in all four survival endpoints we have provided a graphical representation of methods in Figure 8.

It can be seen in Figure 8, diverse types of methods that have been utilized in survival predictive pipelines can be categorized into three different categories i.e., statistical, ML, and DL. Statistical methods are broadly classified into three different categories i.e., parametric, semi-parametric, and non-parametric models. Parametric methods make assumptions about the survival time distribution (Lee and Wang, 2003; Kubi et al., 2022). Parametric methods include exponential, Weibull, log-normal, Weibull, gamma models, and so on (Ishak et al., 2013; Kubi et al., 2022). Comparatively, semi-parametric methods make no assumptions about the shape of the baseline hazard function (non-parametric) (Kleinbaum and Klein, 1996). Rather, these methods assume a specific functional form for the effect of covariates (parametric) (Sinha and Dey, 1997). In comparison, non-parametric methods do not take into account assumptions about the underlying distribution of survival times and the shape of the hazard function. These methods include Kalpan-Meier, Nelson-Aalen, Breslow, Gehan-Eilcoxon, and life table methods (Stevenson and EpiCentre, 2009). Some statistical methods (i.e., COX-PH) have certain disadvantages with multiomics based survival prediction (Lee and Lim, 2019). For instance, COX-PH assumes linear relationships among variables and fails to capture complex and non-linear data patterns (Therneau et al., 2000). These methods perform poorly on high dimensional data where the number of features is larger than the number of samples. This specific gap is filled by the emergence of AI based models. Various ML models are utilized for survival analysis such as random survival forest (Ishwaran et al., 2008), and boosting-based methods (Binder and Schumacher, 2008). Shivaswamy et al. (2007), Van Belle et al. (2007), and Khan and Zubek (2008) proposed ranking and regression-based survival SVM for survival prediction while handling right censored data. Particularly, survival SVM is used in three ways for survival prediction i.e., ranking, regression, and combined. Ishwaran et al. (2008) proposed RSF where log-rank test is utilized for the splitting as compared to the Gini impurity of the classical random forest models.

DL methods are utilized in two ways to model survival prediction tasks i.e., continuous and discrete time (Kvamme et al., 2019). Models like CoxCC and time (Kvamme et al., 2019), piecewise constant hazard or PEANN (Fornili et al., 2014), and DeepSruv (Katzman et al., 2018) are utilized for continuous survival time prediction. Whereas, Nnet-survival (Gensheimer and Narasimhan, 2019), Nnet-survival probability mass function (PMF) (Kvamme and Borgan, 2019b), DeepHit and DeepHit Single (Lee et al., 2018), multi-task logistic regression (MTLR) (Yu et al., 2011; Fotso, 2018), and BCESurv (Kvamme and Borgan, 2019a) are utilized to predict survival in a discrete-time setting.

### 5.6 RQ IX: open source tools and libraries potential for development of survival prediction pipelines

Following the objective research question IX, this section summarizes details of open-source libraries and source codes of existing survival predictors. This comprehensive information will facilitate researchers to build upon existing work, fostering a collaborative environment and accelerating the development of robust and effective survival prediction models.

Table 9 presents an overview of open-source survival prediction models. Among the 90 distinct survival prediction studies, only 28 have provided publicly accessible source code. Among these studies, six studies have utilized R (Kantidakis et al., 2020; Li et al., 2021; Redekar et al., 2022; Zhang S. et al., 2022; Ellen et al., 2023; Willems et al., 2023) and 22 have opted for Python (Jiang et al., 2020; Tong L. et al., 2020; Chai et al., 2021a; Hathaway Q. A. et al., 2021; Hira et al., 2021; Malik et al., 2021; Poirion et al., 2021; Xu et al., 2021; Zhang X. et al., 2021; Zhao L. et al., 2021; Wang et al., 2022; Wu and Fang, 2022; Yin et al., 2022; Zhang J. Z. et al., 2022; Benkirane et al., 2023; Fan et al., 2023; Hao et al., 2023; Lang et al., 2023; Manganaro et al., 2023; Moreno-Sanchez, 2023; Palmal et al., 2023; Shetty et al., 2023). A comprehensive analysis of open source codes reveals that a majority of these tools have been developed from scratch without utilizing any specific survival prediction library (Benkirane et al., 2023; Hao et al., 2023; Manganaro et al., 2023; Shetty et al., 2023).

**Table 9:** Summary of open-source survival prediction methods in existing studies.

**References**	**Disease**	**Approach**	**Source code**
Shetty et al. (2023)	Kidney Papillary, Renal Cell Carcinoma (KIRP)	GeneNet	Link
Pellegrini (2023)	Localized prostate cancer	Coherent Voting Network (CVN)	Link
Hao et al. (2023)	GBM, KRCCC, LSCC, BIC	ANNs for binary survival class prediction	Link
Zhang S. et al. (2022)	Ovarian Cancer	DT, RF, and ANN	Link
Manganaro et al. (2023)	Non-small Cell Lung Cancer	Two layer SVM	Link
Benkirane et al. (2023)	Pan-cancer	ANN	Link
Fan et al. (2023)	Pan-cancer	Survival neural network	Link
Willems et al. (2023)	Colorectal cancer	Lasso penalized cox model	Link
Moreno-Sanchez (2023)	Heart failure	Two-way survival prediction	Link
Ellen et al. (2023)	Non-small cell lung cancer	Elastic net and cox proportional hazard model	Link
Yin et al. (2022)	Pan-cancer	CNN and a cox model (CNN-Cox)	Link
Wang et al. (2022)	Neuroblastoma	Deep neural network (DNN)	Link
Zhang J. Z. et al. (2022)	Breast cancer	Loghazard Net	Link
Wu and Fang (2022)	Glioblastoma, ovary and breast cancers	CoxNet	Link
Redekar et al. (2022)	Glioblastoma multiforme	Cox regression	Link
Hathaway Q. A. et al. (2021)	Atherosclerosis	Various models	Link
Xu et al. (2021)	Cardiovascular disease	DeepSur, Cox-PH, RSF	Link
Poirion et al. (2021)	Pan-cancer	Cox-PH model	Link
Zhao L. et al. (2021)	8 cancer subtypes	DeepOmix based on DNN	Link
Chai et al. (2021a)	Bladder cancer	Cox regression, deep cox neural network	Link
Hira et al. (2021)	Ovarian cancer	Cox-PH regression	Link
Tong et al. (2021)	Ovarian, lung, kidney, and pancreatic cancer	Various survival models	Link
Kantidakis et al. (2020)	Liver transplantation	RSF, Cox-PH, PLANN	Link
Jiang et al. (2020)	Lung adenocarcinoma	Cox-nnet	Link
Tong L. et al. (2020)	Breast cancer	DNN and cox proportional hazard model	Link
Li et al. (2021)	Prostate cancer	BLUP	Link
Malik et al. (2021)	Breast cancer	Deep neural network	Link
Zhang X. et al. (2021)	Pan-cancer	Deep Neural network	Link
Kim (2023)	–	Graph Neural networks	–

Approximately 10 different survival prediction packages or libraries have been developed as shown in Table 10. Each library offers a diverse set of preimplemented statistical, ML, and DL survival prediction models. For instance, Pycox (Kvamme et al., 2019) primarily focuses on continuous and discrete DL survival prediction models such as CoxTime, CoxCC, MTLR, and so on. Lifelines (Davidson-Pilon, 2019), scikit-survival (Pölsterl, 2020), and pysurvival (Fotso et al., 2019 ) cover a wide range of statistical and ML survival prediction models like Cox-PH, RSF, survival support vector machine, and gradient boosting survival (Davidson-Pilon, 2019; Fotso et al., 2019; Pölsterl, 2020).

**Table 10:** Survival analysis libraries, models, and evaluation metrics.

**Library**	**Language**	**Models**	**Evaluation metrics**
Scikit-survival (Pölsterl, 2020)	Python	Cox-PH, Penalized Cox-PH, RSF, Kaplan-Meier, Gradient boosting survival, Survival suppport vector machine	Concordance Index (C-index), Integrated Brier Score
Lifelines (Davidson-Pilon, 2019)	Python	Kaplan-MeierFitter, CoxTimeVaryingFitter, Survival regression, Discrete survival models, Piecewise exponential models	Concordance Index (C-index)
Survival (Therneau and Lumley, 2015)	R	Survival regression, Cox-PH, accelerated time failure (AFT) models, Competing risk analysis,	Hazard Ratios, Log-likelihood, Akaike Information Criterion (AIC)
Statsmodels (McKinney et al., 2011)	Python	PHReg, AFT models	Hazard Ratios, Log-likelihood, Akaike Information Criterion (AIC)
Pycox (Kvamme et al., 2019)	Python	Continuous time models such as Cox-Time, CoxCC, PCHazard and DeepSurv, Discrete time models such as Nnet-survival, probability mass function, DeepHit, multitask logistic regression, and BCEsurv	Concordance Index, integrated and administrative Brier Score, time dependent concordance index, negative and integrated bionomial log likelihood
Pysurvival (Fotso et al., 2019 )	Python	CoxPH, RSF, Kaplan-Meier, Survival Support Vector Machine, multitask logistic regression, Parametric models like exponential, Weibull, Gompertz, log logistic, and log normal	Concordance Index (C-index), Integrated Brier Score
flexsurv (Jackson, 2016)	R	Parametric survival models (e.g., Weibull, Exponential)	Hazard Ratios, Log-likelihood, Akaike Information Criterion (AIC)
mlr3proba (Sonabend et al., 2021)	R	Density estimation measures, Cox-PH, flexible spline models, penalized regression, RSF, Van Belle support vector machine, gradient boostinf machine DeepSurv, DeepHit, CoxTime	Houwelingen's β, C-index, time dependent AUC, log-loss, integrated log loss, Brier and integrated Brier score, and Schmid score
rstpm2 (Clements, 2019)	R	Restricted Mean Survival Time (RMST), Cause-specific Hazard Models, Fine-Gray Model (Competing Risks)	IBS, Time-dependent ROC curves, Grays Test for Equality of Cumulative Incidence Functions
Survex (Spytek et al., 2023)	R	Local and global explanations for survival prediction models	None
SurvSHAP (Krzyziński et al., 2023)	Python	Shapely additive explanations for survival prediction models	–

Notably, addressing the lack of interpretability or explainability in the previously discussed libraries, Spytek et al. (2023) introduced Survex. This library allows researchers to analyze the features responsible for a specific event by offering different methods for both local and global explanations of various survival prediction models.

The selection of a specific library is inherently subjective and depends on factors such as the preferred development platform, choice of survival prediction models, and the specific research question in hand. Therefore, recommendations are made based on the number of survival prediction models and evaluation measures each library offers. For Python, Lifelines (Davidson-Pilon, 2019) and Pycox (Kvamme et al., 2019) are recommended, with Lifelines (Davidson-Pilon, 2019) providing a diverse range of statistical and ML models, while Pycox (Kvamme et al., 2019) is specialized in DL models. Additionally, for R, mlr3proba (Sonabend et al., 2021) is recommended, as it offers a variety of statistical and ML models for survival prediction. Ultimately, selecting a library aligned with individual research needs not only streamlines the development process but also contributes to the overall reliability of survival prediction models.

### 5.7 RQ X: strategies for assessing survival predictors: unveiling common evaluation measures

The main objective of this section is to provide a concise overview of research question X, which focuses on the commonly employed evaluation measures for survival predictive pipelines.

Table 11 shows a compilation of 18 distinct evaluation measures that have been commonly used to evaluate survival prediction pipelines. The survival prediction pipelines can be categorized into two distinct classes namely survival outcome prediction (Lynch et al., 2017) and survival prediction (Tarkhan et al., 2021). Details related to these categories is provided in the background section. Out of 18 evaluation measures mentioned in Table 11, a set of 10 evaluation measures have been employed to assess the performance of survival outcome prediction models. In addition to the aforementioned measures, 8 other evaluation measures have been utilized to assess the performance of survival prediction models.

**Table 11:** A summary of evaluation measures used in survival prediction and survival outcome prediction pipelines.

**Task**	**Evaluation measure**	**Count**	**References**	**Advantages**
	C-index	43	Pellegrini, 2023; Qian et al., 2023; Shetty et al., 2023	Robust, measures discriminatory power. Less sensitive to censoring compared to other metrics.
	BS	7	Kantidakis et al., 2020; Hathaway Q. A. et al., 2021; Xu et al., 2021	Measures accuracy of predicted survival probabilities.
	IBS	5	Xu et al., 2021; Jung et al., 2023; Othman et al., 2023	Considers entire survival time distribution.
Survival prediction	Log Rank *P*-value	9	Jiang et al., 2022; Redekar et al., 2022; Benkirane et al., 2023; Zhang et al., 2023	Tests differences in survival experiences.
		DCA	1	Miao et al., 2022	Accounts for clinical consequences.
		Kappa	3	Zheng et al., 2021; Pellegrini, 2023; Shetty et al., 2023	Measures agreement beyond chance.
		TD-ROC	2	Wang J. et al., 2023; Zhang et al., 2023	Time-dependent evaluation of ROC.
	AUC-pval	1	Pellegrini, 2023	Evaluates AUC significance.
	Odds ratio	1	Pellegrini, 2023	Measures association between groups.
	Likelihood Ratio	1	Tong D. et al., 2020	Helps in understanding the odds of a predicted event occurring compared to the odds of it not occurring.
	AUC-ROC	21	Hao et al., 2023; Pellegrini, 2023; Qian et al., 2023	Provides a comprehensive view of the model's performance across various threshold values.
	Accuracy	12	Hao et al., 2023; Lee et al., 2023	Simple and easy to understand, providing an overall measure of correct predictions.
	Precision	6	Chauhan et al., 2023; Hao et al., 2023; Othman et al., 2023; Wang J. et al., 2023	Useful when the cost of false positives is high, as it focuses on the accuracy of positive predictions.
	Recall	6	Chauhan et al., 2023; Hao et al., 2023; Othman et al., 2023; Wang J. et al., 2023	Emphasizes the ability of the model to capture all positive instances, important for sensitive scenarios.
Survival outcome prediction	MCC	1	Othman et al., 2023	
		F1-Score	2	Zeng et al., 2021; Wang et al., 2022	Harmonizes precision and recall, making it useful when there is a trade-off between false positives and false negatives.
		PPV	1	Zeng et al., 2021; Chauhan et al., 2023	Focuses on the proportion of true positives among positive predictions, providing insights into prediction accuracy.
		NPV	1	Zeng et al., 2021; Chauhan et al., 2023	Focuses on the proportion of true negatives among negative predictions, providing insights into prediction accuracy.

In survival prediction category based evaluation measures, the objective is to capture two distinct characteristics namely, calibration and discrimination (D'Agostino and Nam, 2003; Simino, 2009). Specifically, calibration refers to how well the predicted probabilities of survival align with the actual observed survival rates over time (D'Agostino and Nam, 2003). Under this paradigm most widely used evaluation measures are BS (Schumacher et al., 2003), IBS (Gerds and Schumacher, 2006), TD-ROC (Heagerty et al., 2000), and DCA (Vickers and Elkin, 2006). Discrimination paradigm based evaluation measures capture differentiation between individuals with different survival times. Under this paradigm most widely used measures are C-index (Hartman et al., 2023), AUC-ROC (Terrematte et al., 2022), and likelihood ratio (Murphy, 1995).

On the other hand objective of survival outcome prediction evaluation measures is to assess diverse characteristics of a model i.e., efficacy of the model, overall accurate predictions, biasness toward type I or type II errors (Hao et al., 2023; Lee et al., 2023). Specifically, accuracy and F1 score are used to measure overall accurate predictions, precision, and recall examine the model's biasness with respect to type I and type II errors (Zeng et al., 2021; Wang et al., 2022). Additionally, MCC provides a comprehensive assessment, taking into account overall accurate predictions, and errors (Othman et al., 2023). In addition, AUC-ROC assesses the predictive potential of a model by analyzing the true positive rate (TPR) and true negative rate (TNR) at different thresholds (Hao et al., 2023; Pellegrini, 2023; Qian et al., 2023).

### 5.8 RQ XI: publisher and journal-wise distribution of research papers

This section addresses research question XI by presenting the distribution of survival prediction literature across diverse journals and publishers. Overall, this analysis not only enables researchers to strategically position their work but also offers opportunities for interdisciplinary collaboration, promoting a more interconnected and dynamic research landscape within the domain of survival prediction.

**Figure 9:**
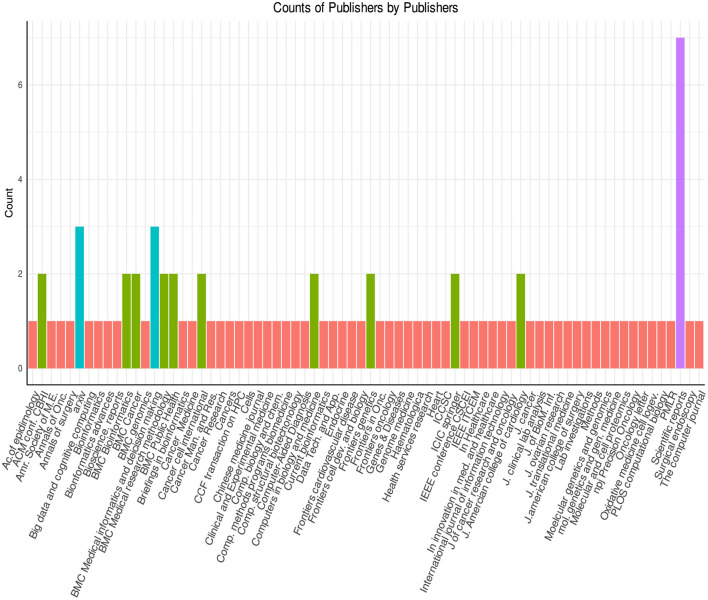
Journal-wise distribution of articles.

**Figure 10:**
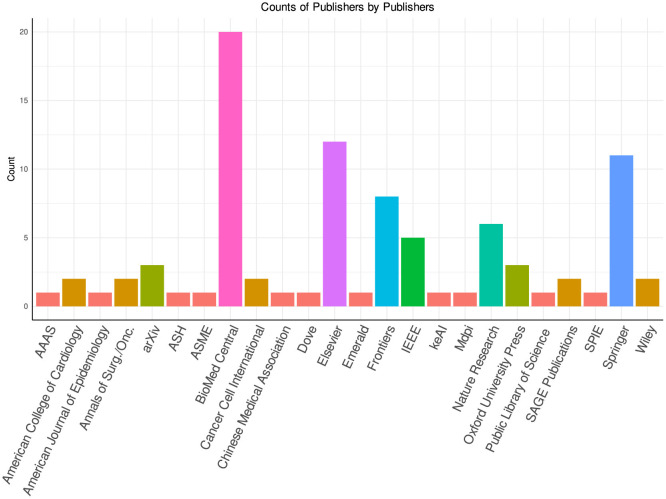
Publisher-wise distribution of articles.

In Figures 9, 10, the distribution of survival prediction literature is presented based on journals and publishers. The studies have been published in 25 different publishers, including but not limited to Springer, Elsevier, Oxford Press, and BioMed Central. Notably, around 30 out of 90 survival prediction studies have been disseminated through Springer, and BioMed Central. Furthermore, Elsevier has contributed to the field by publishing 10 relevant papers in recent years. Particularly, these studies have been published in more than 50 different conferences/journals, which shows the diversity of the survival prediction landscape.

## 6 Discussion

The field of disease survival prediction has become a pivotal aspect of effective healthcare, especially within the domain of precision medicine. Recognizing the significant variability present among patients within specific diseases, there is an increasing demand and development for disease specific survival predictors. Our analysis reveals that researchers place a profound emphasis on predicting survival in cancer as compared to other diseases, and there are compelling reasons behind this focus. First, cancer exhibits significant variability from one patient to another as compared to other diseases, which highlights the imperative need for cancer survival prediction to explore and comprehend the heterogeneity of cancer. Second, cancer is a leading cause of death worldwide, and effective survival prediction can aid in early detection and intervention, potentially saving lives. Third, a huge amount of data sources are developed to make cancer-related data publicly available to accelerate and optimize cancer-related research.

Furthermore, to analyze the trajectory of the disease, researchers place great focus on studying different survival endpoints that suit the respective research setting i.e., treatment, progression, recurrence, and death. Among four different survival endpoints i.e., OS, DFS, BC, and PFS, OS is often emphasized more in survival prediction studies. Despite the prime focus on OS, the significance of other survival endpoints in understanding disease trajectories cannot be understated. These survival endpoints help to analyze different characteristics of diseases such as understanding treatment efficacy and durability, treatments that not only extend life but also effectively manage the course of the illness, and markers responsible for disease recurrence. The lack of research in other survival endpoints opens up new research avenues for the AI experts to develop novel methods that can help explore various characteristics related to disease.

Although both public and private databases have been utilized in survival prediction studies, yet the preference for public databases stems from their accessibility and the wealth of information they provide. For instance, TCGA (Tomczak et al., 2015) offers a vast array of genomic and clinical data across different cancer types. This invaluable resource aids researchers in developing accurate survival prediction models. Likewise, GDC (Jensen et al., 2017) and GEO (Clough and Barrett, 2016) offer comprehensive datasets that encompass a wide range of diseases, making them appealing choices for various research endeavors. Furthermore, a crucial observation regarding private data sources is that they are not universally accessible. This argument is supported by the limited accessibility of omics datasets related to cardiovascular diseases. Despite a singular study employing omics data for survival prediction in cardiovascular diseases, the challenge lies in the difficulty of retrieving the original data. Authors often refrain from sharing their datasets, and obtaining access to databases requires extensive proposals, adding a layer of complexity to the development of novel survival prediction pipelines for cardiovascular diseases. This obstacle may impede the advancement of innovative survival prediction pipelines for cardiovascular disease.

Overall, the use of omics and clinical data in survival prediction tools marks a significant stride toward precision medicine. The distribution of omics types in survival prediction studies reveals a preference for mRNA, methylation, microRNA, and CNV across various cancer subtypes. In addition, the limited number of multiomics based survival prediction studies in cardiovascular diseases hinders definitive conclusions on the importance of specific omics types. Disease-specific patterns highlight the importance of tailored clinical markers, prominently seen in cancer studies with a focus on tumor stage and histological type. Treatment-related features, notably chemotherapy and radiotherapy, underscore the impact of therapeutic interventions on survival predictions. Moreover, clinical features along with omics data with diverse molecular aspects are utilized together to improve the performance of survival prediction models. Diverse survival prediction research accentuates the pivotal role of leveraging patient information, such as medical history, demographics, disease-related features, and diagnostic records. This trend reflects an increasing recognition of the potential of clinical data in not only understanding disease progression but also in guiding personalized treatment strategies and enhancing patient care. A recent benchmark study on survival prediction models with multiomics and clinical data also shows the significant role of clinical data in survival prediction across multiple cancer subtypes (Herrmann et al., 2021).

In addition, our analysis reveals that increasing the total number of data modalities does not necessarily offer improved survival predictions, yet data modalities are quite specific to the disease and survival endpoints. Therefore, the selection of data modalities should be made very carefully as rather than improving the overall performance it can induce undesirable noise in the analysis.

One of the common problems in survival analysis is data censoring (Leung et al., 1997). Censoring arises when there is incomplete information about the time points and/or events of some subjects in a study. There are different types of censoring i.e., (I) Right Censoring is the most common type of data censoring, where an event does not occur for some subjects by the end of study or by the last time point at which data is collected. For example, a subject withdraws from the study or there is a lost follow up for a specific subject (II) Left Censoring is the least common type of censoring where the event may occur before the start of the study or during the data collection phase. (III) Interval Censoring arises when the event of interest occurs in a time interval but the exact time point is not known. In survival analysis, three assumptions are taken into account to infer censored data i.e., (I) Independent Censoring: assumes that the censoring times for multiple subjects are independent of each other. (II) Random censoring assumes that the time t at which individuals are censored must be random and the failure rate for subjects who are censored is assumed to be equal to the failure rate for subjects who remained in the risk set who are not censored. (III) Non-informative censoring occurs if the distribution of survival times (T) provides no information about the distribution of censorship times (C), and vice versa. Although, data censoring is quite important in terms of survival prediction, yet it has been discussed and dealt with properly in the existing studies. We recommend to incorporate comprehensive details of data censoring in future survival prediction studies. Particularly details on how each type of data censoring is handled should not be neglected.

Our analysis of the utilization of feature engineering methods raises two crucial points. First, even though a plethora of methods have been already tested for various survival prediction studies, autoencoder based methods tend to reduce the dimensionality of omics data modalities more efficiently. In addition, the rest of the methods work much better with clinical features. The success of feature engineering approaches is contingent upon the chosen technique with the inherent properties of the data. This highlights the importance of large-scale benchmark studies in guiding the selection of feature engineering strategies for the development of accurate predictive pipelines.

In end-to-end survival predictive pipelines, researchers have utilized methods from three different families namely statistical (Hazard models, Kaplan-Meier Estimator, Log-Rank Test, and Frailty Models) (Kleinbaum and Klein, 1996), ML (Random Forests, Support Vector Machines, Gradient Boosting Machines, and Nearest Neighbors) (Ishwaran et al., 2008; Ma et al., 2022), and DL (CoxNnet, DeepSurv) (Ching et al., 2018; Katzman et al., 2018). Statistical methods are unable to extract complex non-linear patterns that is why in current predictors focus of researchers is on ML or DL based methods (Katzman et al., 2018). In spite of the applications and usefulness of traditional ML methods, they face numerous limitations when applied to survival prediction. These limitations arise either from the inherent challenges of survival data or from the models themselves. Such limitations include censored observations (Khan and Zubek, 2008), overfitting and outliers (Biccler et al., 2020; Nariya et al., 2023), and complex relationships among variables. ML models also suffer from outliers in survival prediction datasets (Biccler et al., 2020). DL methods address many of these limitations through their advanced architectures and ability to learn complex patterns from large datasets. DL models such as DeepSurv, extend the Cox proportional hazards model by learning non-linear representations of the covariates and handling censored data effectively (Katzman et al., 2018). This model leverages the strengths of neural networks to capture complex relationships and interactions between variables, improving prediction accuracy (Katzman et al., 2018). In addition, there are some advantages of ML methods as well, i.e., they perform better even on small datasets while DL methods require large data (LeCun et al., 2015). Similarly, ML methods decisions are explainable and DL methods decisions are black box (Dwivedi et al., 2023). Although, a research comunity is focusing on unveiling black box decisions of predictors. However, in survival prediction, most of predictors do not have explainability component (Krzyziński et al., 2023). But researchers are trying to incorporate explainability methods with survival models (Krzyziński et al., 2023).

While developing different data modalities based on survival predictor, predictive pipelines require dimensionality reduction methods that avoid the curse of the dimensionality problem (Feldner-Busztin et al., 2023). Although several traditional methods (PCA, LDA, TSNE, UMAP etc.) have been developed to transform data into new space that have more comprehensive patterns and less number of features. However, these methods lacks in extracting and incorporating non-linear patterns of features (Gastinel, 2012; Kirpich et al., 2018; Degenhardt et al., 2019). On the other hand in deep learning based predictive pipelines, researchers are utilizing auto-encoders that are capable of generating more comprehensive feature space by extracting both linear and non-linear patterns of features (Tan et al., 2020). Following overall pros and cons of ML and DL based predictive pipelines, new predictors can be developed by utilizing ML based methods with smaller datasets. Moreover, in these predictors rather than utilizing traditional dimensionality reduction methods, autoencoders can be utilized. Moreover, when data is large, it is better to develop DL predictors but these predictors must be enriched with explainability methods.

With an aim to evaluate the performance of predictive pipelines, diverse types of evaluation measures have been developed. Each evaluation measure addresses a specific aspect of survival prediction models, precluding the possibility of any single metric being universally ideal for a comprehensive evaluation of survival prediction. For instance, C-index estimates the robustness and discriminatory power of the survival prediction model. In addition, BS and IBS measure the accuracy of a model on time distribution. Moreover, log-rank p-value evaluates the potential of the model by testing the differences in different survival groups. Although these measures are the most commonly utilized, there are diverse other evaluation measures for similar purposes i.e., restricted mean survival time (RMST), odds ratio (Pellegrini, 2023), Kappa for inter-rater reliability (Zheng et al., 2021), integrated absolute error (IAE), integrated square error (ISE), mean absolute error (MAE), integrated AUC (IAUC) time-dependent integrated discrimination improvement, and time-dependent net reclassification improvement (NRI). Furthermore, while these individual measures provide valuable insights, it is noteworthy to mention that their collective application offers a more comprehensive evaluation. Therefore, we recommend utilizing multiple evaluation measures to assess discrimination and calibration of survival prediction models.

## 7 Reccomendations

With an aim to expedite and enhance research in survival prediction. Hereby, on the basis of Table 12, we summarize some important recommendations for future survival prediction studies.

**Table 12:** A summary of key research questions of our review and main findings.

**Research question (RQ)**	**Main findings**
RQ I, II, III: Survival predictors distribution analysis across diseases and survival endpoints	A detailed analysis of the past three years shows that survival prediction models have been developed majorly for 36 distinct cancer subtypes, as outlined in Table 3. Additionally, the primary focus of this research has been on overall survival (OS) as the clinical/survival endpoint.
RQ IV: Survival prediction data availability in public and private sources and opportunities for the development of predictors	Publicly accessible data primarily comes from three key databases: the Cancer Genome Atlas Program (TCGA), NCI Genomic Data Commons (GDC), and the Gene Expression Omnibus (GEO). In contrast, private databases also exist but present several challenges. Limited accessibility to private data hinders the reproducibility and validation of survival models, and the lack of standardized access complicates model comparison, potentially introducing bias and impacting generalizability. In addition, our recommendation is to use Survboard datasets for benchmarking and testing of survival models, which are available at https://survboard.vercel.app/.
RQ V, VI: Survival prediction data modalities and utilization of their combinations for disease and survival endpoints specific predictors development	The selection of data modalities depends on the disease and survival endpoint. For example, in cancer subtypes, mRNA, miRNA, and methylation data are most frequently used. Similarly, these modalities are commonly employed for OS endpoint, while distinct patterns are less evident for DFS, PFS, and BC endpoints. In addition, clinical features such as demographic, histological, lifestyle, and comorbidity are of high importance and are used commonly in survival analysis.
RQ VII: Feature engineering trends across data modalities and disease-specific survival predictors	In terms of cardiovascular diseases, univariate analyses, and supervised feature engineering methods i.e., Cox regression, L1 regularized Cox regression, and RSF algorithm, are common, while cancer research based on multiomics data opts for dimensionality reduction methods like PCA, AEs, and VAEs. There is no consistent or apparent effect of survival endpoints on the selection of feature engineering method in the published literature.
RQ VIII: Survival Prediction Methods Insights and Distribution Across Disease Types and Survival Endpoints	A plethora of statistical, machine ML, and DL based approaches have been utilized for survival prediction. However, our analysis reveals that statistical methods have significant disadvantages: they depend on strong assumptions like the proportionality of hazards, struggle with high-dimensional data, and complex non-linear relationships. Additionally, they are sensitive to outliers and missing data. In contrast, DL methods such as DeepSurv, Survival Convolutional Neural Networks (SurvCNN), BCESurv, SurNnet, and autoencoders are preferred due to their ability to handle high-dimensional data, model non-linear relationships, and integrate heterogeneous data sources, making them more robust and accurate for multiomics based survival prediction.
RQ IX: Open source tools and libraries potential for development of survival prediction pipelines	For survival analysis in Python, Lifelines and Pycox are commonly used packages. Lifelines provides a diverse range of statistical and ML models, while Pycox specializes in DL models. Alternatively, mlr3proba is also used which can be a good choice for R users, offering a variety of statistical and ML models for survival prediction.
RQ X: Strategies for assessing survival predictors: unveiling common evaluation measures	In our findings on survival prediction evaluation, we discovered two key assessment paradigms: calibration and discrimination. Calibration evaluates the alignment between predicted and observed survival rates over time using measures like BS, IBS, TD-ROC, and DCA. Meanwhile, discrimination measures such as C-index, AUC-ROC, and likelihood ratio focus on distinguishing individuals with varying survival times. Additionally, for overall model evaluation, we identified accuracy, F1 score, precision, recall, MCC, and AUC-ROC as crucial metrics for assessing efficacy, prediction accuracy, and bias toward type I or type II errors.
RQ XI: Publisher and journal-wise distribution of research papers	Our analysis of the survival prediction literature reveals a diverse distribution across various publishers. Among the 16 publishers identified, notable contributions come from Springer, Elsevier, Oxford Press, and BioMed Central. Around 30 out of 74 studies have been disseminated through Springer and BioMed Central, highlighting their significant presence in the field. Additionally, Elsevier has contributed 10 relevant papers in recent years. These studies have been published across more than 50 different conferences and journals, underscoring the breadth and diversity of the survival prediction landscape.

We highly recommend leveraging open-source tools and libraries for developing survival prediction pipelines. Pycox (Kvamme et al., 2019), Lifelines (Davidson-Pilon, 2019), and scikit-survival (Pölsterl, 2020) are excellent choices, offering a rich array of pre-implemented statistical, ML, and DL models. In addition, selecting appropriate evaluation measures is paramount. We advise researchers to carefully choose measures aligned with their research question and survival prediction task. Utilizing multiple measures ensures a comprehensive assessment of model performance i.e., C-index, IBS, BS, and ROC (Schumacher et al., 2003; Gerds and Schumacher, 2006; Terrematte et al., 2022; Hartman et al., 2023).

Integration of clinical and omics data is key to improving prediction accuracy. Researchers should explore diverse data sources and consider disease-specific patterns and survival endpoints to enhance the predictive power of their models. Researchers should carefully use feature engineering methods tailored to their data characteristics. Autoencoder-based dimensionality reduction for omics data and traditional methods for clinical features can significantly enhance predictive pipelines. Particularly it is important to note that addressing data censoring transparently is essential for model reliability. We recommend providing comprehensive details on censoring types and handling methods to ensure the robustness of survival prediction models.

Both traditional ML and DL methods offer unique advantages. Researchers should explore the strengths of each approach, with a particular focus on DL methods like DeepSurv for capturing complex relationships. In addition, models like Transformers can also be used to deal with clinical data which shall be an interesting research perspective in future (Pant et al., 2023). Enriching survival prediction models with explainability methods is crucial for improving interpretability. By understanding and unveiling model decisions, researchers can enhance trust and adoption in clinical settings. By following such recommendations, researchers can contribute to the development of robust and effective survival prediction models, ultimately facilitating personalized treatment strategies and improving patient care across various disease.

## Data availability statement

The original contributions presented in the study are included in the article/Supplementary material, further inquiries can be directed to the corresponding author.

## Author contributions

AA: Conceptualization, Data curation, Formal analysis, Investigation, Methodology, Software, Validation, Visualization, Writing – original draft, Writing – review & editing. MA: Conceptualization, Data curation, Investigation, Methodology, Software, Supervision, Validation, Writing – original draft, Writing – review & editing. SA: Supervision, Writing – review & editing. SV: Supervision, Visualization, Writing – original draft, Writing – review & editing. AD: Supervision, Writing – review & editing.
