# Supplementary material for: Survival prediction landscape: an in-depth systematic literature review on activities, methods, tools, diseases, and databases
Source: Front Artif Intell. 2024 Jul 3;7:1428501. doi: 10.3389/frai.2024.1428501 (PMC11252047; doi:10.3389/frai.2024.1428501)
Supplement: Supplementary file 1 [file Data_Sheet_1.PDF]

Survival Prediction Landscape: An In-Depth Systematic Literature Review on Activities, Methods, Tools, Diseases, and Databases Supplementary Information

Supplementary Information

EDITED BY

AHTISHAM FAZEEL ABBASI
MUHAMMAD NABEEL ASIM
SHERAZ AHMED
SEBASTIAN VOLLMER
ANDREAS DENGEL

Deutsches Forschungszentrum für Künstliche Intelligenz (DFKI), Germany[1]
Rheinland-Pfälzische Technische Universität Kaiserslautern-Landau (RPTU), Germany[2]

2023
FRONTIERS IN ARTIFICIAL INTELLIGENCE

Table of Contents

1 Survival prediction studies and their respective details. 2
2 Distribution of data modalities across survival prediction studies. 12
3 Survival endpoints with respect to survival prediction algorithms and studies. 14
4 Description of diverse survival prediction studies and relevant survival prediction models. 16
5 Survival prediction evaluation measures. 24
6 Journal-wise distribution of articles. 27
7 Publisher-wise distribution of articles. 29

Table 1. Survival prediction studies and their respective details.

	Disease	Feature Engineering	Approach	Dataset Type	Source Code	Database	Evaluation
1	Atherosclerotic cardiovascular disease (ASCVD)	L1 regularized Cox regression, RSF algorithm, and Cox regression	Cox regression, Lasso-cox regression, and random survival forest	Clinical	Contact authors for datasets and models	None	AUC and C-index
2	Kidney Papillary, Renal Cell Carcinoma (KIRP)	Network-Based Stratification (NBS) for data integration	-	-	Link	Broad Firehose, GDAC Link, GDC Link	-
3	Localized prostate cancer	None	e Coherent Voting Network (CVN)	Multiomics and clinical	Link	UCSC Xena and cbioportal	AUC, Kappa, AUC-pval
4	gastrointestinal cancer	None	DeepSurv, Multitask logistic regression (L-MTLR), Gompertz model, Random survival forests (RSF)	Clinical	NS	Data from heidelberg hospital	Integrated Brier score, C-Index, AUC
5	Tripple negative breast cancer	None	Cox and Least Absolute Shrinkage and Selector Operation (LASSO) regression model.	Multiomics	NS	TCGA, GEO, cBioportal	Kaplan Meier analysis and Receiver Operating Characteristic (ROC) analysis

6	Glioblastoma multiforme (GBM), Kidney renal clear cell carcinoma (KRCCC), Lung squamous cell carcinoma (LSCC), and Breast invasive carcinoma (BIC)	ANNs for feature integration	ANNs for binary survival class prediction (long vs short)	Multiomics	Link	TCGA Link	Accuracy, precision, recall, AUC, and Kalpan Meier curve analysis
7	bladder cancer	None	Cox-regression and kalpan meier	Clinical and cell free DNA based omics	NS	Experimentally generated data	None
8	lymphoma	None	Lasso cox regression	Clinical	NS	NS	NS
9	Colon Cancer	DEOD method for data integration	Logistic regression	Clinical and multi-omics	NS	Experimental and GEO datasets Link	AUC, precision, recall
10	hepatocellular carcinoma	Weighted correlation network analysis (WGCNA)	RandomForest, Stepwise Cox (StepCox), Logistic, SurvivalSVM and Lasso	Multiomics	NS	TCGA	C-index
11	Ovarian Cancer	Cox regression analysis and principal component transformation (PCT)	DT, RF, and ANN	Clinical and Multi-omics	Link	TCGA	C-index
12	Non-small Cell Lung Cancer	None	Two layer SVM	Clinical and Multi-omics	Link	TCGA	C-index
13	Pancancer	Variational autoencoders for dimensionality reduction	ANN	Multiomics		e Genomic Data Commons (GDC) and TCGA	C-index, IBS, log rank p-value accuracy,

14	Breast Cancer	None	CNN, LSTM, GRU	Clinical and Multi-omics	NS	TCGA Link	AUC, Accuracy, Precision, Recall, and MCC
15	Lung Adeno-carcinoma	Hierarchal variational autoencoders (H-VAE)	Cox-PH	Clinical and Multiomics data	NS	GEO: GSE81089, GSE63805, and GSE31210	C-index, and Log p-rank values
16	Pancancer	-	-	-	-	-	-
17	Pancancer	ANN with attention mechanism	Survival neural network	Clinical and Multi-omics	Link	TCGA	C-index
18	Colorectal cancer	None	Lasso penalized cox model	Multiomics	Link	TCGA	C-index
19	Heart failure	Filters, wrapper, and embedded feature selection methods: ANOVA, chi-squared, mutual information and recursive feature elimination	Two-way survival prediction: Classification and time prediction. Cox proportional hazard model, random survival forests for survival prediction. Authors also used methods for the interpretability of their model like partial dependence plot, permutation feature extraction, and shapely values.	Clinical	Link	-	C-index, Accuracy, sensitivity, specificity, AUC, and MCC
20	HER2-negative metastatic breast cancer	Canonical correlation analyses	Cox regression	Clinical	NS	Experimental	Time-dependent receiver operating curves (TD-ROC), AUC, and Harrel C-index

21	Pancreatic cancer caused by malignant biliary obstruction	Univariate analyses	Cox-PH regression	Clinical	NS	COMBO-01	AUC
22	Non-small cell lung cancer	Denoising autoencoder	Elastic net and cox proportional hazard model	Multiomics and clinical	Link	TCGA	C-index, accuracy and AUC
23	Trauma Patients	a Least Absolute Shrinkage and Selection Operator (LASSO) regression modeling	RF, SVM	Biomarkers data	Link	None	–
24	Nasopharyngeal Carcinoma	None	Cox-regression and Kalpan Meier	Clinical	NS	Chongqing University Cancer Hospital tumor database	C-index, AUC, Decision curve analysis (DCA)
25	Hypersensitive patients suffering from cardiovascular diseases	NS	Linear multitask logistic regression, neural multitask logistic regression, random survival forest and cox proportional hazard model	Clinical	NS	[26,27]	Concordance index, Brier score, root mean squared error, mean absolute error
28	Pancancer	Cascaded Wx	CNN and a cox model (CNN-Cox)	Multiomics	Link	TCGA	C-index
29	COVID-19	NS	Support vector machine	Multiomics	NS	Experimental	AUC
30	Neuroblastoma	Recursive feature elimination and Patient similarity networks	Deep neural network (DNN)	Multiomics	Link	SEQC and TARGET projects	Accuracy, AUC, and F1-score

31	Breast cancer	CANDECOMP PARAFAC decomposition using alternating least squares and autoencoder	Loghazard Net, a discrete-time survival net based on partial logistic regression	Multiomics	Link	TCGA	C-index and integrated Brier score
32	Invasive ductal carcinoma (type of breast cancer)	NS	Multivariate Cox two way stepwise regression	Whole slide images, clinical and transcriptomic data	NS	TCGA, Molecular signature database (MsigDB)	AUC
33	Stomach and Esophageal carcinoma (STES) and Ovarian serous	NS	Bidirectional long short-term memory, ordinal Cox model network and auxiliary loss	Multiomics and Clinical	NS	TCGA	C-index
34	Lower grade glioma	NS	Lasso cox regression	Multiomics and imaging data	Link	TCGA	AUC, C-index, Brier score
35	Clear cell renal cell cancer (ccRCC)	Boruta and PCA	Cox regression	Multi-omics	NS	TCGA	Log-rank p value, AUC, and C-index
36	Hepatocellular carcinoma	NS	Cox-PH regression	Multiomics	NS	TCGA	NS
37	Glioblastoma, ovary and breast cancers	Autoencoder	CoxNet	Multiomics	Link	TCGA	C-index
38	Ovarian cancer	FALSE	Cox-PH model	Multiomics	NS	GEO (GSE14764, GSE17260, GSE19829, GSE30161, GSE49997, GSE63885)	–
39	Glioblastoma multifrome	NS	Cox regression	Multiomics and clinical	Link	TCGA	Akaike information criterion, Bayesian information criterion, and C-index

40	Cardiovascular disease (CVD)	Akaike Information Criterion (AIC) based stepwise regression analysis	Survival outcome prediction based on naive bayes, artificial neural network, and support vector machine.	Clinical	NS	Kaggle	AUC, accuracy, precision, recall, F1-score
41	Cardiovascular disease (CVD)	NS	Logistic regression and XGboost for survival outcome prediction and cox regression, survival XGboost, and DeepHit neural network for the prediction of time to event based survival.	Clinical and proteomics	NS	Data formulated through LIFE-cohort[42].	C-index, AUC, sensitivity, specificity, positive predicted value (PPV) and negative predicted value (NPV).
43	Atherosclerosis	NS	Cox proportional model, random survival forest, multi-task logistic regression, deepsurv neural network, and linear support vector machines	Clinical, biomarkers, and images	Link	Multi-Ethnic Study of Atherosclerosis (MESA)	C-index
44	Cardiovascular disease	Multi-colinearity	DeepSur, Cox-PH, RSF	Biomarkers, clinical and images	Link	Clinical Practice Research Datalink (CPRD)	C-index and integrated Brier score
45	Cervical cancer	iCluster algorithm	Random forest and cox-PH models	Multi-omcis	NS	TCGA	C-index

46	Pancancer	Inverse rank transformation for normalization, Autoencoder for dimensionality reduction, Kruskal-Wallis for important feature identification and Guassian clustering	Cox-PH model	Multiomics	Link	TCGA and GDC	C-index, log p-rank
47	8 cancer subtypes: stomach adenocarcinoma, ovarian serous cystadenocarcinoma, kidney renal clear cell carcinoma, lower grade glioma, head and neck squamous cell carcinoma, bladder urothelial carcinoma, and breast cancer	Feature removal with small variance, and normalization with min-max scaling	DeepOmix based on deep neural network	Multiomics and clinical	Link	TCGA	C-index
48	Bladder cancer	Autoencoder, PCA, and hierarchal clustering	Cox regression, deep cox neural network (CoxNet), and transfer learning-based CoxNet	Multiomics		TCGA	C-index

49	Bladder cancer	Non-negative matrix factorization (NMF), univariate Cox regression, lasso analysis, and multivariate regression	Random survival forest	Multiomics	NS	ArrayExpress, TCGA, and GEO	-
50	Heart disease	Multi-bloc partial least squares	Cox regression	Multiomics	NS	Experimental data from the University of Illinois at Chicago (UIC)	C-index
51	Hepatocellular carcinoma	Autoencoder	CoxNet	Multiomics	NS	TCGA	Silhouette score
52	Ovarian cancer	Variational autoencoders (VAEs), and maximum mean discrpancy VAEs	Cox-PH regression	Multiomics	Link	TCGA	C-index, Brier score, and AUC
53	Ovarian, and lung cancer, kidney renal cell and lung squamuos cell carcinoma and pancreatic adenocarcinoma	PCA and unsupervised univariate feature selection by variance	Cox-PH, Cox-Time, and DeepSurv with consensus training	Multiomics		TCGA	C-index, and accuracy
54	Liver transplantation	NS	Random survival forest, Cox-PH model, and partial logistic artificial neural networks (PLANN)	Clinical	Link	UNOS	C-index, Brier score, and integrated Brier score

55	Colon adenocarcinoma	Autoencoder, PCA, ANOVA, non-negative matrix factorization	Cox-PH regression	Multiomics	NS	TCGA	Silhouettte index, and Calinski-Harabasz criterion for clustering. Log-rank p-values, C-index, and Brier score for survival prediction.
56	Gastric cancer	NS	LASSO, univariate and multi-variate Cox-PH regression	Multiomics	NS	GEO and TCGA (GSE15459, TCGA-GC, GSE84437)	C-index
57	Lung adninocarcinoma	Variational and multiview factorization autoencoders	Cox-nnet	Multiomics	Link	TCGA	C-index
58	Esophageal squamous cell carcinoma	Univariate cox regression, and autoencoder	Support vector machine, K-means clustering	Multiomics	NS	ArrayExpress and TCGA	Log rank P-value, C-index, Brier score
59	Adult diffuse glioma	NS	Cox regression	Multiomics	NS	TCGA	Harrell C-statistic
60	Liver cancer	Univariate feature selection	XGBoost for subtype classification, and Cox-ph for survival prediction	Multiomics	NS	TCGA and GEO	Log-rank p value and C-index
61	Colon cancer	NS	Random forest	Multiomics	NS	UCSC Xena browser	C-index
62	Pancreatic cancer	Autoencoder	SVM, logistic regression. l2 regularized regression and random forest	Multiomics and clinical	NS	TCGA	C-index and integrated Brier score
63	Breast cancer	Variational autoencoders	Deep neural network along with cox proportional hazard model	Multiomics	Link	TCGA	C-index

64	Colon cancer	Hierarchical clustering	Multi-covariate Cox PH	Multiomics and clinical	NS	TCGA	C-index, likelihood ratio, score and Wald test
65	Pancancer	Autoencoders	Deep neural network	Multiomics	NS	TCGA	AUC and C-index
66	Lung adenocarcinoma	Autoencoder and univariate cox-ph model	LASSO cox-ph regression	Multiomics	NS	TCGA	Log-rank p-value and x-index
67	Glioblastoma multifrome	NS	SVM and Cox-PH	Clinical	radiomics and genomics	NS	NS
68	Glioblastoma multifrome	Univariate cox	LASSO penalized cox regression	Multiomics	NS	TCGA and CCGA	FALSE
69	Prostate cancer	NS	Best Linear Unbiased Prediction (BLUP)	Multiomics and clinical	Link	TCGA	NS
70	Non-small-Cell Lung Cancer	ResNet	Deep neural network	SENet	EfficientNet	and ResNeXt	Multiomics and clinical
71	Breast cancer	Neighborhood component analysis (NCA)	Deep neural network	Multiomics	Link	TCGA	Sensitivity
72	Breast cancer	Variance and Chi-squared	ResNet	Multiomics	NS	TCGA	Accuracy
73	Pancancer	Variational autoencoder	Deep Neural network	Multiomics	Link	Genomic Data Commons (GDC) pan-cancer multi-omics dataset and the DNA methylation dataset of human central nervous system tumours (GSE109381)	Macro-F1
74	Pancancer	NS	Lasso or Group Lasso-penalized Cox model	Multiomics	NS	TCGA	Kappa

Table 2. Distribution of data modalities across survival prediction studies.

Study	Clinical	mRNA	miRNA	Methylation	CNV	Proteomics	Mutation	Metabolic	lncRNA	WES
Pellgrini et al. 2023[3]	Yes	Yes	No	Yes	No	Yes	No	No	No	No
Jun et al. 2023[4]	Yes	No	No	No	No	No	No	No	No	No
Qian et al. 2023[1]	Yes	No	No	No	No	No	No	No	No	No
Chauhan et al. 2023[7]	Yes	No	No	No	No	No	No	No	No	No
Li et al. 2023[8]	Yes	No	No	No	No	No	No	No	No	No
Moreno et al. 2023[19]	Yes	No	No	No	No	No	No	No	No	No
Wang et al. 2023[20]	Yes	No	No	No	No	No	No	No	No	No
Zhou et al. 2023[21]	Yes	No	No	No	No	No	No	No	No	No
Zhang et al. 2023[5]	No	No	Yes	No	No	No	No	No	No	No
Hao et al. 2023[6]	No	Yes	Yes	Yes	No	No	No	No	No	No
Lee et al., 2023[9]	Yes	Yes	No	No	Yes	No	No	No	No	No
Wang et al. 2023[10]	No	Yes	No	No	No	No	No	No	No	No
Zhan et al. 2022[11]	Yes	Yes	No	No	Yes	No	Yes	No	No	No
Manganaro et al. 2023[12]	Yes	Yes	No	No	Yes	No	Yes	No	No	No
Benkirane et al. 2023[13]	No	No	No	No	No	No	No	No	No	No
Othman et al. 2023[14]	Yes	Yes	No	No	Yes	No	No	No	No	No
Bhat et al. 2023[15]	Yes	Yes	Yes	Yes	Yes	No	No	No	No	No
Fan et al. 2023[17]	Yes	Yes	Yes	No	Yes	No	No	No	No	No
Willems et al. 2023[18]	No	Yes	Yes	No	No	No	No	No	No	No
Ellen et al. 2023[22]	Yes	Yes	Yes	Yes	No	No	No	No	No	No
Yin et al. 2022[28]	No	Yes	No	No	No	No	No	No	No	No
Richard et al. 2022[29]	No	No	No	No	No	Yes	No	Yes	No	No
Wang et al. 2022[30]	No	Yes	No	Yes	No	No	No	No	No	No
Zhang et al. 2022[31]	No	Yes	No	Yes	No	No	No	No	No	No
Miao et al. 2022[24]	Yes	No	No	No	No	No	No	No	No	No
Lin et al. 2022[32]	Yes	No	No	No	No	No	No	No	No	No
Feng et al. 2022[25]	Yes	No	No	No	No	No	No	No	No	No
Bichindaritz et al. 2022[33]	Yes	Yes	No	Yes	No	No	No	No	No	No

Wu et al. 2022[34]	No	Yes	No	Yes	No	No	No	No	No	No
Jiang et al. 2022[35]	No	Yes	Yes	Yes	No	No	No	No	Yes	Yes
Zhang et al. 2022[36]	No	Yes	No	No	Yes	No	No	No	No	No
Wu et al. 2022[37]	No	Yes	No	No	Yes	No	No	No	No	No
Hathaway et al. 2021[43]	Yes	No	No	No	No	No	No	No	No	No
Pawar et al. 2022[38]	No	Yes	No	No	No	No	No	No	No	No
Redekar et al. 2022[39]	Yes	Yes	No	Yes	Yes	No	No	No	No	No
Han et al. 2022[75]	No	Yes	No	Yes	Yes	No	Yes	No	No	No
Zeng et al. 2021[40]	Yes	No	No	No	No	No	No	No	No	No
Unterhuber et al. 2021[41]	Yes	No	No	No	No	Yes	No	No	No	No
Hu et al. 2021[45]	No	Yes	Yes	Yes	Yes	No	Yes	No	No	No
Poirion et al. 2021[46]	No	Yes	Yes	Yes	No	No	No	No	No	No
Kantidakis et al. 2020[54]	Yes	No	No	No	No	No	No	No	No	No
Zhao et al. 2021[47]	Yes	Yes	Yes	Yes	Yes	No	No	No	No	No
Chai et al. 2021[48]	No	Yes	Yes	Yes	Yes	No	No	No	No	No
Tang et al. 2021[49]	No	Yes	No	No	No	No	No	No	No	No
Vahabi et al. 2021[50]	No	Yes	No	Yes	No	No	No	No	No	No
Malik et al. 2021[71]	No	Yes	No	Yes	Yes	Yes	Yes	No	No	No
Owens et al. 2021[51]	No	Yes	Yes	Yes	No	No	No	No	No	No
Hira et al. 2021[52]	No	Yes	Yes	Yes	Yes	No	No	No	No	No
Tong et al. 2021[53]	No	Yes	Yes	Yes	Yes	No	No	No	No	No
Du et al. 2020[68]	No	Yes	No	No	Yes	No	No	No	No	No
Li et al. 2021[69]	Yes	Yes	Yes	Yes	No	No	No	No	No	No
Zhang et al. 2021[70]	No	Yes	Yes	Yes	No	No	No	No	No	No
Zhou et al. 2021[72]	No	Yes	No	No	Yes	No	No	No	No	No
Zhang et al. 2021[73]	No	Yes	Yes	Yes	No	No	No	No	No	No
Zheng et al. 2021[74]	No	No	Yes	No	Yes	No	Yes	No	No	No
Li et al. 2020[56]	No	Yes	No	Yes	No	No	No	No	No	No
Kazerooni et al. 2021[67]	Yes	No	No	No	No	No	No	No	No	No
Lv et al. 2020[55]	No	Yes	Yes	Yes	No	No	No	No	No	No
Jiang et al. 2020[57]	No	Yes	Yes	Yes	Yes	No	No	No	No	No
Yu et al. 2020[58]	No	Yes	No	Yes	No	No	No	No	No	No

Yang et al. 2020[59]	No	Yes	No	No	No	No	Yes	No	No	No
Wang et al. 2020[60]	No	Yes	Yes	Yes	No	No	No	No	No	No
Yang et al. 2020[61]	No	Yes	Yes	Yes	No	No	No	No	No	No
Baek et al. 2020[62]	Yes	Yes	Yes	Yes	No	No	No	No	No	Yes
Tong et al. 2020[63]	No	Yes	Yes	Yes	Yes	No	No	No	No	No
Tong et al. 2020[64]	Yes	Yes	Yes	Yes	No	No	No	No	No	No
Tan et al. 2020[65]	No	Yes	Yes	Yes	No	Yes	No	No	No	No
Lee et al. 2020[66]	No	Yes	Yes	Yes	Yes	No	No	No	No	No

Table 3. Survival endpoints with respect to survival prediction algorithms and studies.

Study	Models	Survival Endpoint
Qian et al. 2023[1]	Cox-PH, RSF, MTLR, deepsurv neural network	OS
Shetty et al. 2023[2]	GeneNet	OS
Pellegrini et al. 2023[3]	Coherent Voting Network (CVN), Best Linear Unbiased Prediction (BLUP)	DFS, OS, BC
Jung et al. 2023[4]	DeepSurv, MTLR, RSF, Gompertz model	OS
Zhang et al. 2023[5]	Lasso Cox-PH	OS
Hao et al. 2023[6]	ANNs with custom loss	OS
Chauhan et al. 2023[7]	Cox-PH, RSF, CoxNet, transfer learning-based CoxNet PFS, OS	
Li et al. 2023[8]	Lasso-Cox-PH	OS
Lee et al. 2023[9]	Loghazard Net, partial logistic regression, Cox-PH	DFS, OS, BC
Wang et al. 2023[10]	Stepwise Cox (StepCox), SurvivalSVM, Cox-PH, CoxNet	OS
Manganaro et al. 2023[12]	SVM, Elastic net and Cox-PH, CNN and ANN	DFS
Benkirane et al. 2023[13]	ANN	OS
Othman et al. 2023[14]	CoxNet, Cox-PH, Cox-Time, DeepSurv with consensus training, Loghazard Net, partial logistic regression	OS
Bhat et al. 2023[15]	CoxNet, Cox-PH, Cox-Time, DeepSurv with consensus training, Loghazard Net, partial logistic regression	OS
Majji et al. 2023[16]	Survival neural network, CNN-Cox, Cox-PH, DeepOmix, lasso and group penalized Cox-PH, VAE based NN	DFS
Fan et al. 2023[17]	CNN-Cox, Cox-PH, DeepOmix, lasso and group penalized Cox-PH, VAE based NN	OS

Willems et al. 2023[18]	Lasso Cox-PH	OS
Wang et al. 2023[20]	Cox-PH	PFS, OS
Zhou et al. 2023[21]	Cox-PH, l2 regularized regression	OS
Ellen et al. 2023[22]		OS
Abdelhamid et al. 2022[23]	RF, SVM	OS
Miao et al. 2022[24]	Cox-regression	OS
Feng et al. 2022[25]		OS
Yin et al. 2022[28]	CNN-Cox	OS
Richard et al. 2022[29]	SVM	OS
Wang et al. 2022[30]		OS
Zhang et al. 2022[31]	DL algorithm with three-dimensional tensor representation	OS
Lin et al. 2022[32]	Multivariate Cox models	OS
Bichindaritz et al. 2022[33]	Bidirectional long short-term memory, ordinal Cox model network and auxiliary loss	OS
Wu et al. 2022[34]	LASSO Cox-PH	OS
Jiang et al. 2022[35]	Cox-PH	PFS, OS
Zhang et al. 2022[36]	Cox-PH	OS
Wu et al. 2022[37]	SAEsurv-net	OS
Pawar et al. 2022[38]	Cox Proportional Hazard model	OS
Redekar et al. 2022[39]	Cox-PH	OS
Han et al. 2022[75]	OS	
Zhang et al. 2022[11]	OS	
Unterhuber et al. 2021[41]	Various ML and DL models	OS
Hathaway et al. 2021[76]		OS
Xu et al. 2021[44]	Various models	OS
Hu et al. 2021[45]	Cox-PH	OS
Poirion et al. 2021[46]	DeepProg	OS
Zhao et al. 2021[47]	DNN with custom loss	OS
Chai et al. 2021[48]	TCAP	OS
Tang et al. 2021[49]		DFS, PFS, OS
Vahabi et al. 2021[50]	Cox-sMBPLS	OS, BC

Table 4. Description of diverse survival prediction studies and relevant survival prediction models.

Study	Approach	Description
Qian et al., 2023[1]	Cox-PH and RSF	A study to predict survival of ASCVD patients in Xinjiang population based on ML and statistical models based on clinical data with a follow up time of 5.79 years.
Shetty et al., 2023[2]	GeneNet	GeneNet based survival prediction in KIRP based on multiomics data.
Pellegrini et al., 2023[3]	Coherent Voting Network (CVN)	A CVN model selection based survival prediction model for localized prostate cancer on the basis of clinical and multiomics data.
Jung et al., 2023[4]	DeepSurv, Multitask logistic regression (L-MTLR), Gompertz model, Random survival forests (RSF)	AI based experimentation on the cllinical datasets of gastrointestinal cancer generated from Heidellberg university hospital.
Zhang et al., 2023[5]	Cox and Least Absolute Shrinkage and Selector Operation (LASSO) regression model.	This study employed Cox and ASSO regression models to construct a survival model for triple-negative breast cancer (TNBC). By clustering mRNA expression profiles from TTCGA based on tumor immune microenvironment, the resulting risk score model was validated across databases and clinical samples, offering a reliable tool for predicting TNBC prognosis and guiding immunotherapy efficacy.
Hao et al., 2023[6]	ANNs with custom loss	The study addresses the challenge of heterogeneous cancer data by proposing a deep learning approach for survival prediction. Integrating mRNA expression, DNA methylation, and microRNA expression data, the model represents each genetic data type with shared and specific features, enhancing its ability to capture consensus and complementary information. Experimental results across four cancers show the approach significantly outperforms established integrative methods, providing an effective tool for accurate cancer survival prediction. Particularly, it performs a multiclass classificaiton task of predictting survival in different classes on four multiomics based datasest of GBM, KRCCC, LSCC, and BIC.

Chauhan et al., 2023[7]	Cox-PH and Kalpan meier	It employed ultra-low-pass whole genome sequencing (ULP-WGS) and urine cancer personalized profiling by deep sequencing (uCAPP-Seq) to analyze urine tumor DNA in 74 localized bladder cancer patients.
Li et al., 2023[8]	Lasso Cox-PH	The study developed a prognostic model for lymphoma patients' overall survival (OS) through LASSO regression and Cox stepwise regression analyses, incorporating nine independent risk factors. The nomograms exhibited good prediction accuracy (C-indices: 0.749 in training, 0.731 in validation), and calibration and ROC curves validated the model's reliability for 1-year, 3-year, and 5-year OS predictions.
Lee et al., 2023[9]	Logistic regression	This study utilized machine-learning analysis to identify survival-related candidate genes for colon cancer, leading to the identification of RABGAP1L, MYH9, and DRD4. A survival prediction model, combining these genes with tumor stages, demonstrated superior predictive performance in assessing the prognosis of colon cancer patients.
Wang et al., 2023[10]	RandomForest, StepCox, Logistic, SurvivalSVM and Lasso	LncRNA based analysis of survival across hepatocellular patients on the basis of WGCNA and 5 ML survival prediction models.
Zhang et al., 2022[11]	DT, RF, and ANN	5-year overal survival prediction across ovarian cancer patients. Initially, optimal features were identified using RFI and RSF.
Manganaro et al., 2023[12]	SVM	This study introduced mtSVM, a novel computational approach for survival prediction in NSCLC using data from the PROMOLE clinical study. mtSVM, a two-layer feed-forward network of FastSurvivalSVMs, outperforms other methods, exhibiting excellent performance in risk stratification based on clinical and multiomics data.
Benkirane et al., 2023[13]	ANN	The paper explored deep learning methods for integrating multi-omics data, focusing on autoencoders and their effectiveness in cancer-related tasks across diverse datasets.The model with alternate AE training stretgy and SHAP demonstrated strong performance in cancer-related tasks across diverse TCGA datasets, providing interpretable results for tumor classification and survival outcome prediction.

Othman et al., 2023[14]	CNN, LSTM, GRU	This study addressed the challenges of early breast cancer detection by proposing a hybrid deep learning model based on CNN for feature extraction and GRU and LSTM for classification. The model used clinical, gene expression, and copy number alteration data, and made predictions on breast cancer.
Bhat et al., 2023[15]	Cox-PH	A deep hierarchal variational autoencoder (H-VAE) model was developed for joint analysis of multi-omics data, identifying two distinct patient subgroups with significant survival differences. The integraiton model was used with Cox-Ph for survival prediction
Majji et al., 2023[16]		SSDH-based DRNN, a method developed for predicting cancer-survival rates using gene expression data. The approach employed polynomial kernel data transformation, Bayesian fuzzy clustering for gene selection, and survival indicators like SMA and rate of change, achieving high accuracy on the Pan-Cancer dataset. Trained with SSDH, a combination of SSA and DHOA, the method demonstrated its potential for precise cancer survival prediction and clinical management.
Fan et al., 2023[17]	Survival neural network	This work aimed to enhance cancer survival prediction using multi-omics data by constructing a deep learning model with multimodal representation and integration. The unsupervised learning part extracted high-level features from diverse omics data, while an attention-based method integrated these representations into a compact vector for survival prediction. Particularly, this worked utilized pancancer survival datasets.
Willems et al., 2023[18]	LASSO Cox-PH	In addressing colorectal cancer challenges, this study introduced a novel metric validated for its association with miRNA-gene target interactions. Employing this metric with a regularized Cox model, the study identified a small set of top-performing genes linked to colon cancer, demonstrating a significant improvement in survival prediction and accurate patient risk stratification.
Moreno et al., 2023[19]	Cox-PH, random survival forests for survival prediction	In addition to survival prediction, authors also used methods for the interpretability of their model like partial dependence plot, permutation feature extraction, and shapely values.

Wang et al., 2023[20]	Cox-PH	A clinical data based multi observational study on HER2-negative metstatic breast cancer based on Cox-regression
Zhou et al., 2023[21]	Cox-PH	A clinical data based first of its kind survival prediciton model for ”Pancreatic cancer caused by malignant biliary obstruction” by using Cox-regression.
Ellen et al., 2023[22]	Elastic net and Cox-PH	Multiomics and clinical data based model to predict non-small cell lung cancer (NSCLC) survival by using Cox-PH. Denoising autoencoders were employed for data compression and integration, and performance was compared across modality combinations and data integration methods for lung adenocarcinoma (LUAD) and squamous cell carcinoma (LUSC) using TCGA data. Results indicate that survival prediction models combining multiple modalities outperformed single-modality models, with the highest performance achieved using a combination of lncRNA and clinical data .
Abdelhamid et al., 2022[23]	RF, SVM	Biomarkers based prediction of important features for 30 day survival outcome prediction in Tarauma patients based on RF and SVM.
Miao et al., 2022[24]	Cox-PH and Kalpan-Meier	OS prediction based on clinical features of a chinese cohort suffering from nasophrngeal carcinoma. The work utilized cox-regression for survival prediction.
Feng et al., 2022[25]	Linear multi-task logistic regression, neural multi-task logistic regression, random survival forest and Cox-PH	Clinical data based comparative analysis of survival models for the prediction of incident hospitlization in cardiovascular patients.
Yin et al., 2022[28]	CNN and a cox model (CNN-Cox)	The study introduced the CNN-Cox model, a concise and efficient survival analysis model, which combined CNN with Cox-PH, achieving superior performance across seven cancer types in TCGA cohort on the basis of multiomics data.
Richard et al., 2022[29]	SVM	Outcome prediction in COVID-19 patients based on SVM and concentration values of 10 proteins and 5 metabolites.
Wang et al., 2022[30]	Deep neural network (DNN)	Outocome prediciton in neuroblastoma patients by incorporating multiomics data with PSNs.

Zhang et al., 2022[31]	Loghazard Net, a discrete-time survival net based on partial logistic regression	DL algorithm with three-dimensional tensor representation to integrate three types of omics data for improved cancer survival prediction in breast and colon cancers.
Lin et al., 2022[32]	Multivariate Cox two way stepwise regression	Survival outcome prediction in terms of invasive ductal carcinoma based on whole slide images, clinical and biomakers data. These features are passed through multivariate Cox models at different steps.
Bichindaritz et al., 2022[33]	Bidirectional long short-term memory, ordinal Cox model network and auxiliary loss	The study proposed an approach for cancer survival prediction, utilizing bidirectional long short-term memory, an ordinal Cox model network, and auxiliary loss, merging gene expression and DNA methylation data for ovarian and esopageal carcinoma.
Wu et al., 2022[34]	Lasso Cox-PH	Risk strification and survival prediciton based on multiomics and imaging data for lower grade glioma with LASSO Cox-PH
Jiang et al., 2022[35]	Cox-PH	OS and PFI prediction for clear cell renal cell carcinoma based on multiomics data and Cox-PH.
Zhang et al., 2022[36]	Cox-PH	To address the poor prognosis of hepatocellular carcinoma (HCC), a multi-omics analysis was conducted . A prognostic model based on Cox-PH incorporating genomic variants and prognosis-related genes identified 78 candidates, with a 5-gene signature (CISH, LHPP, MGMT, PDRG1, and LCAT) established through random forest feature selection.
Wu et al., 2022[37]	CoxNet	This paper introduced SAEsurv-net, a novel method for cancer survival prediction through multi-omics data integration, addressing challenges like high dimensionality and heterogeneity. SAEsurv-net employs a two-stage dimensionality reduction strategy and a stacked autoencoder model to achieve a balance between computation complexity and information exploitation while handling heterogeneities in the data. The survival prediction head of the overall pipeline was CoxNnet model.
Pawar et al., 2022[38]	Cox-PH	It aimed to establish gene signatures for better prognosis in ovarian cancer patients. Using the Cox Proportional Hazard model, 20 upregulated and 20 downregulated genes were selected for overall analysis.

Redekar et al., 2022[39]	Cox-PH	Survival prediction in terms of glioblastoma with Cox-PH mode based on multiomics and clinical data modalities.
Zeng et al., 2021[40]	Survival outcome prediction based on naive bayes, artificial neural network, and support vector machine.	Survival outcome prediction in a binary classificaiton setting using NB, ANNs and clinical data obtained through Kaggle for cardiovascular diseases.
Unterhuber et al., 2021[41]	Logistic regression, XGboost and Cox-PH, survival XGboost, and DeepHit neural network.	Prediction of survival time and outcome based on various ML and DL models by utllizing proteomics and clinical data for cardiovascular diseases.
Hathaway et al., 2021[43]	Cox proportional model, random survival forest, multi-task logistic regression, deepsurv neural network, and linear support vector machines	The study aimed to enhance atherosclerosis risk prediction using ML and DL survival models. On the basis of MESA data, the DeepSurv model significantly improved ASCVD risk prediction compared to other models. The study demonstrated that DeepSurv effectively leveraged basic clinical features for accurate ASCVD risk prediction without requiring additional inflammatory or imaging biomarkers.
Xu et al., 2021[44]	DeepSur, Cox-PH, RSF	Cardiovascular disease survival prediciton on the basis of diverse biomarkers and clinical features using various models.
Hu et al., 2021[45]	Random forest and cox-PH models	It demsontrated the prediciton of survival prediction in cervical cancer based on multiomics data.
Poirion et al., 2021[46]	Cox-PH model	Authors proposed DeepProg, a novel way of intergrating multiomics data for cancer survival prediction. Particular, the overall architecture is based on ANNs and Cox-PH model for pancancer survival prediction.
Zhao et al., 2021[47]	DeepOmix based on deep neural network	The study focused on creating a DNN based architecture with custom loss to intergrate nnon-linear modalities of multiomics data with survival prediciton at the same point. In addition, the model is evaluated against data of 8 different cancer subtypes.
Chai et al., 2021[48]	Cox regression, deep cox neural network (CoxNet), and transfer learning-based CoxNet	This study introduced TCAP, a transfer-learning based Cox proportional hazards network, predicting bladder cancer prognosis by integrating multi-omics data using autoencoders.

Tang et al., 2021[49]	Random survival forest	-
Vahabi et al., 2021[50]	Cox-PH	The study introduced Cox-sMBPLS, a supervised model for time-to-event data, designed to integrate multimodal Omics data by considering redundant information and leveraging prior biological associations for cardiovascular disease survival prediction
Owens et al., 2021[51]	CoxNet	This study employed a novel ANN with a custom loss to identify critical features in Hepatocellular Carcinoma (HCC) multi-omics data, revealing biologically homogeneous prognostic subgroups and improvements in survival prediction
Hira et al., 2021[52]	Cox-PH	In this study, an integrated multi-omics analysis of ovarian cancer was conducted using Variational Autoencoder (VAE) and Maximum Mean Discrepancy VAE (MMD-VAE) for dimensionality reduction. The DL-based architecture successfully classified transcriptional subtypes with reasonable accuracies.
Tong et al., 2021[53]	Cox-PH, Cox-Time, and DeepSurv with consensus training	The main focus is related to the data integration part, where invariant representations were generated using DNNs and tested for ovarian and breast cancer with multiple survival prediction models.
Kantidakis et al., 2020[54]	Random survival forest, Cox-PH model, and partial logistic artificial neural networks (PLANN)	Clinical data based survival prediction in liver transplantation patients through SNNs, RSF, and PLANN.
Lv et al., 2020[55]	Cox-PH	Auhors aimed to design AE based feature intergation method for multiomics data, and surival was performed using COx-PH for lung adenocarcinoma.
Li et al., 2020[56]	LASSO, univariate and multi-variate Cox-PH regression	Cox-PH based survival prediciton in gastric cancer based on multiomics data.
Jiang et al., 2020[57]	Cox-nnet	TLSurv, a super-hybrid network, utilized multi-stage transfer learning to integrate diverse -omics data for lung cancer survival prediction. Particularly, it is based on multiomics data and CoxNnet

Yu et al., 2020[58]	Support vector machine, K-means clustering	The study leveraged deep learning autoencoder to identify prognosis-related features in esophageal squamous cell carcinoma (ESCC), revealing two distinct risk subgroups with significantly different overall survival rates, paving the way for potential effective diagnostic modalities for early-stage ESCC.
Yang et al., 2020[59]	Cox regression	Overal differential gene expression and survival analysis were carried out on the basis of multiomics data in glioma.
Wang et al., 2020[60]	XGBoost for subtype classification, and Cox-ph for survival prediction	In this study, a method combining deep learning and network fusion was proposed to predict liver cancer survival subtypes using TCGA data. The model identified two subgroups with significant survival differences, validated in GEO cohorts.
Yang et al., 2020[61]	Random forest, and Cox-PH	Clinical outcome prediction was performed using RF and multiomics data for colon cancer. In addition, Cox-PH was applied for survival prediction.
Baek et al., 2020[62]	SVM, logistic regression. l2 regularized regression and random forest, Cox-PH	Survival outcome and survival prediction based on multiomics and clinical features which were integrated via AE.
Tong et al., 2020[63]	Deep neural network along with cox proportional hazard model	AE based data integration of multiomics data and survival prediction through AE based CoxNnet model.
Tong et al., 2020[64]	Multi-covariate Cox PH	Survival prediction in colon cancer with multiple combinations of clinical, and multiomics datasets usign multivariate Cox-PH.
Tan et al., 2020[65]	Deep neural network	MOSAE (Multi-omics Supervised Autoencoder), effectively employed specific autoencoders for individual omics, generated task-specific representations through supervised learning, and achieved superior predictive performance across four clinical outcome endpoints on the TCGA pancancer dataset.
Lee et al., 2020[66]	LASSO cox-ph regression	Authors introduced a deep learning-based autoencoding approach, incorporating multiomics data, resulting in a robust model with significant survival subgroup differentiation and high consistency index for liung adenocarcinoma.

Kazerooni et al., 2021[67]	SVM and Cox-PH	This study showed the significance of integrated diagnostics, combining clinical, radiomic, and genomic data, for enhanced overall survival prediction in newly diagnosed, treatment-naïve, IDH-wildtype glioblastoma patients,
Du et al., 2020[68]	LASSO penalized cox regression	Multiomics based survival prediction in glioblastoma with penalized Cox-PH model.
Li et al., 2021[69]	Best Linear Unbiased Prediction (BLUP)	Genomic Selection (GS) methodology, specifically the Best Linear Unbiased Prediction (BLUP) model, demonstrated superior predictability and computational efficiency for prostate cancer (PCa) prognosis when applied to TCGA data, offering a valuable approach to enhance outcome prediction and address the issue of overtreatment in PCa management.
Zhang et al., 2021[70]	CNN and ANN	NSCLC related outcome and survival prediction based on multiomcis and clinical data.
Malik et al., 2021[71]	Deep neural network	A late multi-omics integrative framework utilizing neighborhood component analysis (NCA) and neural network models effectively quantified survival and drug response for breast cancer patients.
Zhang et al., 2021[73]	VAE based NN	Omnibed utilized VAE based NN for dimensionality reduction and survival prediction based on multiomics data.
Zheng et al., 2021[74]	Lasso or Group Lasso-penalized Cox model	This study systematically assessed the prognostic landscape across 33 cancer types, utilizing multiomics data. The findings showed the cancer type-dependent predictive performance of omics data, with gene expression data consistently offering superior predictive accuracy.

Table 5. Survival prediction evaluation measures.

Publication	Evaluation Measure(s)
[1]	AUC, C-index, Brier score
[2]	-
[3]	AUC, Kappa, AUC-pval, odds ratio
[4]	Integrated Brier score, C-Index, AUC
[5]	Kaplan Meier analysis and Receiver Operating Characteristic (ROC) analysis

6	Accuracy, precision, recall, AUC, and Kalpan Meier curve analysis
7	PPV, NPV, Precision, Recall, hazard ratio
8	NS
9	AUC, precision, recall
10	C-index
11	C-index
12	C-index
13	C-index, IBS, log rank p-value accuracy,
14	AUC, Accuracy, Precision, Recall, and MCC
15	C-index, and Log p-rank values
16	FALSE
17	C-index
18	C-index
19	C-index, Accuracy, sensitivity, specificity, AUC, and MCC
20	Time-dependent receiver operating curves (TD-ROC), AUC, and Harrel C-index
21	AUC
22	C-index, accuracy and AUC
23	–
24	C-index, AUC, Decision curve analysis (DCA)
25	C-index, Brier score, root mean squared error, mean absolute error
28	C-index
29	AUC
30	Accuracy, AUC, and F1-score
31	C-index and integrated Brier score
32	AUC
33	C-index
34	AUC, C-index, Brier score
35	Log-rank p value, AUC, and C-index
36	NS
37	C-index
38	–
39	C-index

40	AUC, accuracy, precision, recall, F1-score
41	C-index, AUC, sensitivity, specificity, positive predicted value (PPV) and negative predicted value (NPV).
43	C-index
44	C-index and integrated Brier score
45	C-index
46	C-index, log p-rank
47	C-index
48	C-index
49	-
50	C-index
51	Silhoutee for clustering
52	C-index, Brier score, and AUC
53	C-index, and accuracy
54	C-index, Brier score, and integrated Brier score
55	Silhouettte index, and Calinski-Harabasz criterion for clustering. Log-rank p-values, C-index, and Brier score for survival prediction.
56	C-index
57	C-index
58	Log rank P-value, C-index, Brier score
59	Harrell C-statistic
60	Log-rank p value and C-index
61	C-index
62	C-index and integrated Brier score
63	C-index
64	C-index, likelihood ratio, score and Wald test
65	AUC and C-index
66	Log-rank p-value and c-index
67	NS
68	FALSE
69	NS
70	FALSE
71	Sensitivity

72	Accuracy
73	Macro-F1
74	Kappa

Table 6. Journal-wise distribution of articles.

Journal	Counts
BMC Public Health	2
Moelcular genetics and genomics	1
Scientific reports	6
J of cancer research and oncology	1
Clinical and Experimental medicine	1
BMC Bioinformatics	2
npj Precision Oncology	1
BMC Medical informatics and deicision making	3
Cancer Medicine	1
BMC Cancer	2
J. clinical lab analysis	1
Current bioinformatics	1
PLOS computational biology	1
Big data and cognitive computing	1
International journal of information technology	1
The computer journal	1
Bionformatics advances	1
Cells	1
Frontiers cardiovascular disease	1
Chinese medicine journal	1
Surgical endoscopy	1
J.american college of surgery	1
Cancer cell international	2
BMC Medical research methodology	2
Heart	1

Health services research	1
Lab investigations	1
Molecular and cell proteomics	1
Bioinformatics	1
Computers in biology and medicine	2
In innovation in med. and healthcare	1
Frontiers oncology	1
Oxidative medicine cell logev.	1
arxiv	3
J. ovarian research	1
Comp. methods programs biomedicine	1
IEEE conference CSCEI	1
J. Amercian college of cardiology	2
American college of epidimology	1
Frontiers genetics	2
Genome medicine	1
Comp. structural biotechonology	1
CCF transaction on HPC	1
J. translational medicine	1
Methods	1
Bioscience. reports	1
ACM conf. CBHI	2
Oncology letter	1
J. cancer	1
mol. genetics and gen. medicine	1
Comp. biology and chem.	1
Frontiers cell dev. and biology	1
Briefings in bioinformatics	1
ICIC springer	2
BMC genomics	1
Cancers	1
IEEE ITCEM	1

Table 7. Publisher-wise distribution of articles.

Publisher	Counts
BioMed Central	20
Springer	9
Nature Research	6
Elsevier	11
Frontiers	7
Public Library of Science	1
Wiley	2
SAGE Publications	2
AAAS	1
Oxford University Press	3
IEEE	4
arXiv	3
Chinese Medical Association	1
Cancer Cell International	2
American College of Cardiology	2
American Journal of Epidemiology	1

References

1. Qian, X. *et al.* Study on the prediction model of atherosclerotic cardiovascular disease in the rural xinjiang population based on survival analysis. *BMC Public Heal.* **23**, 1–11 (2023).
2. Shetty, K. S., Jose, A., Bani, M. & Vinod, P. Network diffusion-based approach for survival prediction and identification of biomarkers using multi-omics data of papillary renal cell carcinoma. *Mol. Genet. Genomics* 1–12 (2023).
3. Pellegrini, M. Accurate prognosis for localized prostate cancer through coherent voting networks with multi-omic and clinical data. *Sci. Reports* **13**, 7875 (2023).
4. Jung, J.-O. *et al.* Machine learning for optimized individual survival prediction in resectable upper gastrointestinal cancer. *J. Cancer Res. Clin. Oncol.* **149**, 1691–1702 (2023).
5. Zhang, J., Zhang, M., Tian, Q. & Yang, J. A novel model associated with tumor microenvironment on predicting prognosis and immunotherapy in triple negative breast cancer. *Clin. Exp. Medicine* 1–15 (2023).
6. Hao, Y., Jing, X.-Y. & Sun, Q. Cancer survival prediction by learning comprehensive deep feature representation for multiple types of genetic data. (2023).
7. Chauhan, P. S. *et al.* Urine cell-free dna multi-omics to detect mrd and predict survival in bladder cancer patients. *npj Precis. Oncol.* **7**, 6 (2023).
8. Li, X. *et al.* Development and validation of prediction model for overall survival in patients with lymphoma: a prospective cohort study in china. *BMC Med. Informatics Decis. Mak.* **23**, 1–11 (2023).
9. Lee, C.-J. *et al.* Machine learning with in silico analysis markedly improves survival prediction modeling in colon cancer patients. *Cancer Medicine* **12**, 7603–7615 (2023).
10. Wang, X. *et al.* Machine learning integrations develop an antigen-presenting-cells and t-cells-infiltration derived lncrna signature for improving clinical outcomes in hepatocellular carcinoma. *BMC cancer* **23**, 1–16 (2023).
11. Zhang, S., Zeng, X., Lin, S., Liang, M. & Huang, H. Identification of seven-gene marker to predict the survival of patients with lung adenocarcinoma using integrated multi-omics data analysis. *J. Clin. Lab. Analysis* **36**, e24190 (2022).
12. Manganaro, L. *et al.* Non-small cell lung cancer survival estimation through multi-omic two-layer svm: A multi-omics and multi-sources integrative model. *Curr. Bioinforma.* **18**, 658–669 (2023).
13. Benkirane, H., Pradat, Y., Michiels, S. & Cournède, P.-H. Customics: A versatile deep-learning based strategy for multi-omics integration. *PLoS Comput. Biol.* **19**, e1010921 (2023).
14. Othman, N. A., Abdel-Fattah, M. A. & Ali, A. T. A hybrid deep learning framework with decision-level fusion for breast cancer survival prediction. *Big Data Cogn. Comput.* **7**, 50 (2023).
15. Bhat, A. R. & Hashmy, R. Hierarchical autoencoder-based multi-omics subtyping and prognosis prediction framework for lung adenocarcinoma. *Int. J. Inf. Technol.* 1–9 (2023).
16. Majji, R., Rajeswari, R., Vidyadhari, C. & Cristin, R. Squirrel search deer hunting-based deep recurrent neural network for survival prediction using pan-cancer gene expression data. *The Comput. J.* **66**, 245–266 (2023).
17. Fan, Z., Jiang, Z., Liang, H. & Han, C. Pancancer survival prediction using a deep learning architecture with multimodal representation and integration. *Bioinforma. Adv.* **3**, vbad006 (2023).
18. Willems, A., Panchy, N. & Hong, T. Using single-cell rna sequencing and microrna targeting data to improve colorectal cancer survival prediction. *Cells* **12**, 228 (2023).
19. Moreno-Sanchez, P. A. Improvement of a prediction model for heart failure survival through explainable artificial intelligence. *Front. Cardiovasc. Medicine* **10** (2023).
20. Wang, J. *et al.* Multi-omics fusion analysis models with machine learning predict survival of her2-negative metastatic breast cancer: a multicenter prospective observational study. *Chin. Med. J.* **136**, 863–865 (2023).
21. Zhou, H.-F. *et al.* Survival prediction for patients with malignant biliary obstruction caused by pancreatic cancer undergoing biliary drainage: the combo-pas model. *Surg. Endosc.* **37**, 1943–1955 (2023).
22. Ellen, J. G., Jacob, E., Nikolaou, N. & Markuzon, N. Autoencoder-based multimodal prediction of non-small cell lung cancer survival. *Sci. Reports* **13**, 15761 (2023).
23. Abdelhamid, S. *et al.* Multi-omic admission-based biomarkers predict 30-day survival and persistent critical illness in trauma patients after injury. *J. Am. Coll. Surg.* **235**, S95 (2022).

24. Miao, S. *et al.* Development and validation of a risk prediction model for overall survival in patients with nasopharyngeal carcinoma: a prospective cohort study in china. *Cancer Cell Int.* **22**, 1–11 (2022).

25. Feng, Y. *et al.* Personalized prediction of incident hospitalization for cardiovascular disease in patients with hypertension using machine learning. *BMC Med. Res. Methodol.* **22**, 1–11 (2022).

26. Quan, H. *et al.* Incidence, cardiovascular complications and mortality of hypertension by sex and ethnicity. *Heart* **99**, 715–721 (2013).

27. Metcalfe, A. *et al.* Case definitions for acute myocardial infarction in administrative databases and their impact on in-hospital mortality rates. *Heal. services research* **48**, 290–318 (2013).

28. Yin, Q., Chen, W., Zhang, C. & Wei, Z. A convolutional neural network model for survival prediction based on prognosis-related cascaded wx feature selection. *Lab. Investig.* **102**, 1064–1074 (2022).

29. Richard, V. R. *et al.* Early prediction of covid-19 patient survival by targeted plasma multi-omics and machine learning. *Mol. & Cell. Proteomics* **21** (2022).

30. Wang, C., Lue, W., Kaalia, R., Kumar, P. & Rajapakse, J. C. Network-based integration of multi-omics data for clinical outcome prediction in neuroblastoma. *Sci. Reports* **12**, 15425 (2022).

31. Zhang, J. Z., Xu, W. & Hu, P. Tightly integrated multiomics-based deep tensor survival model for time-to-event prediction. *Bioinformatics* **38**, 3259–3266 (2022).

32. Lin, Z. *et al.* A multi-omics signature to predict the prognosis of invasive ductal carcinoma of the breast. *Comput. Biol. Medicine* **151**, 106291 (2022).

33. Bichindaritz, I. & Liu, G. Adaptive multi-omics survival analysis in cancer. In *Innovation in Medicine and Healthcare: Proceedings of 10th KES-InMed 2022*, 51–62 (Springer, 2022).

34. Wu, W. *et al.* A novel multi-omics analysis model for diagnosis and survival prediction of lower-grade glioma patients. *Front. Oncol.* **12**, 729002 (2022).

35. Jiang, A. *et al.* Establishment of a prognostic prediction and drug selection model for patients with clear cell renal cell carcinoma by multiomics data analysis. *Oxidative Medicine Cell. Longev.* **2022** (2022).

36. Zhang, R. *et al.* Using integrated multi-omics data analysis to identify 5-gene signature for predicting survival of patients with hepatocellular carcinoma. (2022).

37. Wu, X. & Fang, Q. Stacked autoencoder based multi-omics data integration for cancer survival prediction. *arXiv preprint arXiv:2207.04878* (2022).

38. Pawar, A., Chowdhury, O. R., Chauhan, R., Talole, S. & Bhattacharjee, A. Identification of key gene signatures for the overall survival of ovarian cancer. *J. Ovarian Res.* **15**, 1–13 (2022).

39. Redekar, S. S., Varma, S. L. & Bhattacharjee, A. Identification of key genes associated with survival of glioblastoma multiforme using integrated analysis of tcga datasets. *Comput. Methods Programs Biomed. Updat.* **2**, 100051 (2022).

40. Zeng, W., Wang, X., Xu, K., Zhang, Y. & Fu, H. Prediction of cardiovascular disease survival based on artificial neural network. In *2021 IEEE International Conference on Computer Science, Electronic Information Engineering and Intelligent Control Technology (CEI)*, 219–224 (IEEE, 2021).

41. Unterhuber, M. *et al.* Proteomics-enabled deep learning machine algorithms can enhance prediction of mortality. *J. Am. Coll. Cardiol.* **78**, 1621–1631 (2021).

42. Loeffler, M. *et al.* The life-adult-study: objectives and design of a population-based cohort study with 10,000 deeply phenotyped adults in germany. *BMC public health* **15**, 1–14 (2015).

43. Hathaway, Q. A., Yanamala, N., Budoff, M. J., Sengupta, P. P. & Zeb, I. Deep neural survival networks for cardiovascular risk prediction: The multi-ethnic study of atherosclerosis (mesa). *Comput. Biol. Medicine* **139**, 104983 (2021).

44. Xu, Z. *et al.* Prediction of cardiovascular disease risk accounting for future initiation of statin treatment. *Am. journal epidemiology* **190**, 2000–2014 (2021).

45. Hu, Q. *et al.* A new hpv score system predicts the survival of patients with cervical cancers. *Front. Genet.* **12**, 747090 (2021).

46. Poirion, O. B., Jing, Z., Chaudhary, K., Huang, S. & Garmire, L. X. Deepprog: an ensemble of deep-learning and machine-learning models for prognosis prediction using multi-omics data. *Genome medicine* **13**, 1–15 (2021).

47. Zhao, L. *et al.* Deepomix: A scalable and interpretable multi-omics deep learning framework and application in cancer survival analysis. *Comput. structural biotechnology journal* **19**, 2719–2725 (2021).

48. Chai, H., Zhang, Z., Wang, Y. & Yang, Y. Predicting bladder cancer prognosis by integrating multi-omics data through a transfer learning-based cox proportional hazards network. *CCF Transactions on High Perform. Comput.* **3**, 311–319 (2021).

49. Tang, C., Yu, M., Ma, J. & Zhu, Y. Metabolic classification of bladder cancer based on multi-omics integrated analysis to predict patient prognosis and treatment response. *J. translational medicine* **19**, 1–15 (2021).

50. Vahabi, N. *et al.* Cox-smbpls: An algorithm for disease survival prediction and multi-omics module discovery incorporating cis-regulatory quantitative effects. *Front. Genet.* **12**, 701405 (2021).

51. Owens, A. R., McInerney, C. E., Prise, K. M., McArt, D. G. & Jurek-Loughrey, A. Novel deep learning-based solution for identification of prognostic subgroups in liver cancer (hepatocellular carcinoma). *BMC bioinformatics* **22**, 1–22 (2021).

52. Hira, M. T. *et al.* Integrated multi-omics analysis of ovarian cancer using variational autoencoders. *Sci. reports* **11**, 6265 (2021).

53. Tong, L., Wu, H. & Wang, M. D. Integrating multi-omics data by learning modality invariant representations for improved prediction of overall survival of cancer. *Methods* **189**, 74–85 (2021).

54. Kantidakis, G. *et al.* Survival prediction models since liver transplantation-comparisons between cox models and machine learning techniques. *BMC Med. Res. Methodol.* **20**, 1–14 (2020).

55. Lv, J., Wang, J., Shang, X., Liu, F. & Guo, S. Survival prediction in patients with colon adenocarcinoma via multiomics data integration using a deep learning algorithm. *Biosci. Reports* **40**, BSR20201482 (2020).

56. Li, Y., Sun, R., Zhang, Y., Yuan, Y. & Miao, Y. A methylation-based mrna signature predicts survival in patients with gastric cancer. *Cancer Cell Int.* **20**, 1–10 (2020).

57. Jiang, Y., Alford, K., Ketchum, F., Tong, L. & Wang, M. D. Tlsurv: Integrating multi-omics data by multi-stage transfer learning for cancer survival prediction. In *Proceedings of the 11th ACM International Conference on Bioinformatics, Computational Biology and Health Informatics*, 1–10 (2020).

58. Yu, J., Wu, X., Lv, M. *et al.* A model for predicting prognosis in patients with esophageal squamous cell carcinoma based on joint representation learning. oncol lett 20 (6): 1–10 (2020).

59. Yang, Q. *et al.* Integrating genomic data with transcriptomic data for improved survival prediction for adult diffuse glioma. *J. Cancer* **11**, 3794 (2020).

60. Wang, Z. *et al.* An integration framework for liver cancer subtype classification and survival prediction based on multi-omics data. In *Intelligent Computing Methodologies: 16th International Conference, ICIC 2020, Bari, Italy, October 2–5, 2020, Proceedings, Part III 16*, 247–257 (Springer, 2020).

61. Yang, H. *et al.* A novel prognostic model based on multi-omics features predicts the prognosis of colon cancer patients. *Mol. Genet. & Genomic Medicine* **8**, e1255 (2020).

62. Baek, B. & Lee, H. Prediction of survival and recurrence in patients with pancreatic cancer by integrating multi-omics data. *Sci. reports* **10**, 18951 (2020).

63. Tong, L., Mitchel, J., Chatlin, K. & Wang, M. D. Deep learning based feature-level integration of multi-omics data for breast cancer patients survival analysis. *BMC medical informatics decision making* **20**, 1–12 (2020).

64. Tong, D. *et al.* Improving prediction performance of colon cancer prognosis based on the integration of clinical and multi-omics data. *BMC Med. Informatics Decis. Mak.* **20**, 1–15 (2020).

65. Tan, K., Huang, W., Hu, J. & Dong, S. A multi-omics supervised autoencoder for pan-cancer clinical outcome endpoints prediction. *BMC Med. Informatics Decis. Mak.* **20**, 1–9 (2020).

66. Lee, T.-Y., Huang, K.-Y., Chuang, C.-H., Lee, C.-Y. & Chang, T.-H. Incorporating deep learning and multi-omics autoencoding for analysis of lung adenocarcinoma prognostication. *Comput. Biol. Chem.* **87**, 107277 (2020).

67. Kazerooni, A. F. *et al.* Multi-omic prediction of overall survival in patients with glioblastoma: Additive and synergistic value of clinical measures, radiomics, and genomics. (2021).

68. Du, J. *et al.* Identification of prognostic model and biomarkers for cancer stem cell characteristics in glioblastoma by network analysis of multi-omics data and stemness indices. *Front. cell developmental biology* **8**, 558961 (2020).

69. Li, R. *et al.* Extended application of genomic selection to screen multiomics data for prognostic signatures of prostate cancer. *Briefings Bioinforma.* **22**, bbaa197 (2021).

70. Zhang, Z.-S., Xu, F., Jiang, H.-J. & Chen, Z.-H. Prognostic prediction for non-small-cell lung cancer based on deep neural network and multimodal data. In *Intelligent Computing Theories and Application: 17th International Conference, ICIC 2021, Shenzhen, China, August 12–15, 2021, Proceedings, Part III 17*, 549–560 (Springer, 2021).

71. Malik, V., Kalakoti, Y. & Sundar, D. Deep learning assisted multi-omics integration for survival and drug-response prediction in breast cancer. *Bmc Genomics* **22**, 1–11 (2021).

72. Zhou, L., Rueda, M. & Alkhateeb, A. Identifying biomarkers of nottingham prognosis index in breast cancer survivability. In *Proceedings of the 12th ACM Conference on Bioinformatics, Computational Biology, and Health Informatics*, 1–9 (2021).

73. Zhang, X., Xing, Y., Sun, K. & Guo, Y. Omiembed: a unified multi-task deep learning framework for multi-omics data. *Cancers* **13**, 3047 (2021).

74. Zheng, X., Amos, C. I. & Frost, H. R. Pan-cancer evaluation of gene expression and somatic alteration data for cancer prognosis prediction. *BMC cancer* **21**, 1–11 (2021).

75. Han, M., He, J. & Jiao, X. Research on prognostic risk assessment algorithm based on graph neural networks and attention mechanisms. In *2022 International Conference on Information Technology, Communication Ecosystem and Management (ITCEM)*, 101–106 (IEEE, 2022).

76. Hathaway, Q., Yanamala, N., Budoff, M., Sengupta, P. & Zeby, I. Cardiovascular risk stratification through deep neural survival networks-the multi-ethnic study of atherosclerosis (mesa). *J. Am. Coll. Cardiol.* **77**, 561–561 (2021).
